# Factors influencing post-diarrhea growth patterns among children under two years old in rural Bangladesh

**DOI:** 10.1371/journal.pntd.0014699

**Published:** 2026-11-05

**Authors:** Jade Lewis, Yiyi Zhou, Jamie Perin, Sampa Dash, Mohammad Ali, Malathi Ram, Farina Naz, Eva Sultana, Bharati Roy, A. B. M. Ali Hasan, Farzana Ali Afroze, David A. Sack, Fahmida Tofail, Tahmeed Ahmed, A. S. G. Faruque, Subhra Chakraborty

**Affiliations:** 1 Department of International Health, Johns Hopkins Bloomberg School of Public Health, Baltimore, Maryland, United States of America; 2 International Centre for Diarrhoeal Disease Research, Bangladesh, icddr, b, Dhaka, Bangladesh; 3 Kumudini Women’s Medical College and Hospital, Mirzapur, Tangail, Bangladesh; Institut Pasteur, FRANCE

## Abstract

Undernutrition in children is a major global health issue. Diarrhea exacerbates undernutrition, creating a vicious cycle of infection and malnutrition. This longitudinal study examined growth patterns and influencing factors among 138 children following diarrhea. Children under two seeking care for watery diarrhea at a rural hospital in Bangladesh were enrolled and followed for three months of home-based visits. Anthropometric measurements—length-for-age z-score (LAZ), weight-for-age z-score (WAZ), weight-for-length z-score (WLZ), and mid-upper arm circumference (MUAC) were taken at enrollment and follow-ups. Linear mixed-effects models assessed associations of clinical, sociodemographic, sanitation, hygiene, and food security factors with growth outcomes. Overall, LAZ declined over three months (-0.260, SD: 0.408), while WAZ, WLZ, and MUAC improved (0.100, SD: 0.484; 0.249, SD: 0.725; 0.672 cm, SD: 0.476). Children with malnutrition at enrollment strongly predicted persistent malnutrition (LAZ < -2, WAZ < -2, WLZ < -2, MUAC < 12.5 cm) during follow-up compared to new incidence of malnutrition (RR: 3.8, p < 0.001, 95% CI: 1.80, 6.35). Poor growth outcomes were linked to longer diarrhea duration, more vomiting, and hospitalization. Hospitalization was associated with declines in LAZ (-0.095, p = 0.009) and MUAC (-0.098, p = 0.030). Children aged 7–12 months had greater declines in WAZ (-0.109, p = 0.096) and WLZ (-0.181, p = 0.113). Improved sanitation and hygiene supported growth, while household hunger was linked to declines. This study highlights the multifactorial nature of post-diarrhea growth outcomes and emphasizes the need for integrated interventions addressing clinical care, sanitation, hygiene, and food security.

## Author summary

Diarrheal diseases remain a major cause of illness among young children in low- and middle-income countries and can contribute to poor growth and undernutrition. While many children recover quickly after diarrhea, others experience lasting nutritional consequences that may affect their health and development. Understanding which children are most vulnerable to growth faltering after a diarrheal episode could help guide interventions and improve recovery. We followed children under two years of age in rural Bangladesh for three months after they sought care for acute watery diarrhea. We examined how clinical characteristics, nutritional status, household conditions, food security, sanitation, and hygiene practices were associated with changes in growth after illness. We found that children who were already malnourished at the time of diarrhea were more likely to remain malnourished during follow-up. Several other factors, including longer diarrhea duration, vomiting, hospitalization, household hunger, and poor sanitation and hygiene conditions, were also associated with poorer growth outcomes. Our findings suggest that preventing growth faltering after diarrhea requires more than treating the acute illness. Interventions that address nutrition, food security, and household environmental conditions may help improve recovery and support healthy child growth in vulnerable populations.

## 1. Introduction

Despite remarkable progress towards the 2025 Global Nutrition Targets [1] and 2030 Sustainable Development Goals (SDG) 2.2 [2] in the past few decades, child undernutrition remains a significant global health challenge, disproportionately affecting children under 5 years old in low- and middle-income countries (LMICs) [3]. According to recent global estimates in 2020 and 2022, 148.1 million (22.3%) children under five years old suffered from stunting, 45.0 million (6.8%) from wasting, and 85.4 million (12.6%) from underweight [4,5]. Notably, 95% of the stunted children and 97% of the wasted children were in Asia and Africa [4]. Accumulated evidence indicates that undernutrition in early life is associated with a higher risk of premature deaths related to infections such as diarrhea, measles, and pneumonia, accounting for 45% of child mortality, and impairs neurobehavioral and cognitive development among those survivors [6–10].

Bangladesh, as a South Asian country, bears one of the highest burdens of child undernutrition. The country has initiated the first and second National Plans of Action for Nutrition, which outlined strategies to eliminate malnutrition in its population. It has achieved impressive declines in childhood undernutrition, as can be seen from a decrease of 50.3% in the prevalence of stunting and another decrease of 38.3% in the prevalence of underweight among children under five between 1991 and 2022 [11–13]. Yet, with 23.6%, 22.3%, and 11% of the children continue to suffer from stunting, underweight, and wasting, respectively, according to the most recent national survey in 2022 [11–13].Undernutrition continues to contribute significantly to child mortality, having been identified as an underlying factor in up to 60% of deaths among children under five, primarily by increasing vulnerability to infectious diseases and impaired immune function [14,15].

Determinants of child undernutrition could be multifold, including food insecurity and poor nutrition, maternal factors (e.g., low maternal education level, poor maternal nutrition, short maternal stature, etc.), household factors (e.g., low household income, size of the family, unimproved sanitation and water supply), inadequate breastfeeding and complementary feeding practices, low birth weight, and severe infections early in life, which varies by population, sites, and demographic factors [16–21]. Although the etiology of child undernutrition is complex, symptomatic and asymptomatic infections by common enteric pathogens have consistently been a pivotal contributor to child undernutrition in LMICs [6,22–26]. A comparative risk assessment analysis across 137 developing countries estimated that diarrhea was responsible for 5.8 million (13.1%) stunted children worldwide [18,22].

While WASH interventions consistently reduce diarrheal incidence in LMICs [27,28], their impact on linear growth remains equivocal. Large, randomized trials – including the WASH Benefits Bangladesh trial [29] and the SHINE trial in Zimbabwe [30] – found that even combined water, sanitation, handwashing, and nutrition interventions had only marginal effects on stunting, despite significant reductions in diarrhea. A mediation analysis of the WASH Benefits Bangladesh trial further revealed that improved sanitation reduced biomarkers of environmental enteric dysfunction but failed to substantially improve linear growth [31], suggesting that non-diarrhea pathways (e.g., chronic inflammation, nutrient malabsorption) may dominate the causal pathway. The MAL-ED study corroborated this, demonstrating that diarrheal burden alone explained less than 5% of growth faltering across diverse settings [32,33]. These findings underscore the need to examine post-diarrheal recovery in context-specific ways, including factors like microbiota composition or repeated subclinical infections [26,32,34,35]. The close link between diarrhea and undernutrition is concerning as undernourished children are more susceptible to gastrointestinal infections and diarrhea, thus falling into a diarrhea-undernutrition-diarrhea cycle [36]. In rural Bangladesh, where this cycle persists despite WASH improvements [29,33], identifying factors that disrupt it – particularly those influencing post-diarrhea growth trajectories – could unlock targeted strategies to mitigate chronic undernutrition.

It is pivotal to break out of this vicious cycle and improve the chronic sequelae in children who frequently experience diarrhea during their first few years of life. However, evidence is limited in rural Bangladesh on the key factors affecting post-diarrhea undernutrition among children. A previous study found that moderate-to-severe diarrhea, children aged 24–59 months, lower mother’s education, and lower household income were significantly associated with undernutrition in children with diarrhea presenting at the hospital [37]. Another study focused on examining environmental risk factors of wasting in children admitted to a hospital in Bangladesh but did not account for the role of diarrhea [38]. Current evidence related to risk factors of child undernutrition in Bangladesh was mostly generated from cross-sectional analyses, while individual factors may change over time, and so do their relationships with undernutrition [38]. Knowledge of how longitudinal history of diarrhea and other important factors affect children’s growth is still lacking.

In this study, we aim to characterize growth patterns following diarrhea and identify key factors influencing post-diarrhea growth trajectory among children under two years old with all-cause watery diarrhea seeking care from a tertiary level hospital in rural Bangladesh. This analysis was guided by a conceptual framework based on WHO and UNICEF models of child undernutrition, distinguishing between distal sociodemographic factors, environmental exposures, and proximal clinical factors influencing post-diarrheal growth.

## 2. Methods

### 2.1. Ethics statement

Caregivers of all study participants reviewed and signed informed consent. The institutional review boards of the International Center for Diarrheal Disease Research, Bangladesh (icddr,b) and Johns Hopkins Bloomberg School of Public Health approved the study protocol (IRB number: 00015285).

### 2.2. Study description and study population

#### 2.2.1. Study site.

Mirzapur is a rural sub-district (Upazila) of Bangladesh that covers 374 Square km in Tangail district. It is located 60 km northwest of the capital city, Dhaka. Kumudini Women’s Medical College and Hospital is a 750-bed nonprofit hospital located in the central urban union of Mirzapur was established in 1938 to provide health services to the surrounding poor rural population. Since 1982, a diarrhea treatment unit was created and serves nearly 1,500 diarrhea patients each year for treatment. The Kumudini Hospital provides free treatment to all those who are unable to afford medical care.

#### 2.2.2. Study description.

Children under two years old with any watery diarrhea seeking care at the Kumudini Hospital were enrolled in the ongoing study Impact of Non-Dysentery Shigella Infection on the Growth and Health of Children over Time (INSIGHT) in Bangladesh and followed for three months from enrollment [39].

#### 2.2.3. Inclusion and exclusion criteria.

The inclusion criteria of the study population were children > 1 month and < 24 months of age seeking care at Kumudini Hospital for watery diarrhea, residing within the catchment area, and willing to be available for sample and data collection during the scheduled visits. Exclusion criteria included diarrhea started more than 48 hours before enrollment, having already taken antibiotics for diarrhea in the past three days or having antibiotics prescribed for current diarrhea, presenting with another significant disease process requiring specific therapy, presenting with severe acute malnutrition (i.e., WLZ < -3) or bilateral oedema. Children who only completed the baseline anthropometry measurements or were withdrawn from the study before the second anthropometry measurement were excluded from the analysis. We restricted the analysis to children with watery diarrhea to maintain a clinically homogeneous population and align with the parent INSIGHT study design focused on non-dysenteric diarrheal illness. Children with severe acute malnutrition (SAM) were excluded due to the ethical and clinical management considerations, as these children require specialized therapeutic interventions that may independently influence growth trajectories.

### 2.3. Data collection

Socio-demographic information, diarrhea morbidity data, sanitation and hygiene, and food security information were collected by administering questionnaires to caregivers of the study children at the baseline visit. Diarrhea morbidity data were collected every other day following the baseline visit till one week and then every two days until day 90 via a questionnaire administered to the caregivers. Anthropometry of the children was measured at the baseline, followed by days 14, 30, 60, and 90. Medical records were reviewed to identify any hospitalization for diarrhea illness.

### 2.4. Outcome assessment

The continuous outcomes of interest were changes in length-for-age z score (LAZ), weight-for-length z score (WLZ), weight-for-age z scores (WAZ), and mid-upper arm circumference (MUAC) between baseline and each follow-up anthropometry measurement. Enrollment anthropometry measurement of WAZ, WLZ, and MUAC occurred after rehydration therapy was given to the children. The binary outcomes of interest were four indicators of undernutrition (MUAC <12.5 cm), including stunting (i.e., LAZ < -2), wasting (WLZ < -2), and underweight (WAZ < -2), using the WHO reference population [7,40]. Anthropometry z scores were calculated based on the WHO Child Growth Standards using the WHO-issued “Anthro” package in R software [41].

### 2.5. Risk factors

Several clinical, socio-demographic, sanitation and hygiene, and food security factors were examined in this analysis, guided by a conceptual framework informed by WHO and UNICEF models of child undernutrition and a directed acyclic graph (DAG) developed to distinguish confounders, mediators, and time-varying exposures (S1 Fig) [42–44] and previous literature [45–47].

Based on this framework, baseline characteristics such as age, sex, baseline nutritional status, maternal education, and household income were treated as potential confounders. Environmental factors such as sanitation, hygiene, and food security were conceptualized as intermediate exposures, while clinical indicators, including diarrhea duration, vomiting, and hospitalization, were considered proximal factors potentially mediating the relationship between environmental conditions and growth outcomes.

The clinical factors included were the severity of diarrhea (based on the Modified Vesikari Severity Score) [27], number of stools in the last 24 hours of enrollment, duration of diarrhea, vomiting and fever since last visit, dehydration status at enrollment (based on the WHO guidelines for dehydration), hospitalization due to diarrhea, and anorexia at enrollment (defined as the loss of appetite).

The sanitation and hygiene factors included are the type of soap found in the household for handwashing (based on whether the field worker visually confirmed soap in the household), reported handwashing before preparing food and feeding children, and toilet facility type. Under toilet facility, improved sanitation was defined as facilities separating human waste from human contact, flush/pour-flush to piped sewer system, ventilated-improved pit latrines, or pit latrines with slab or composting toilets; unimproved sanitation contains shared toilet facilities, flush/pour-flush not to piped sewer system, pit latrines without slabs, bucket or hanging latrines, and open defecation [45].

The food security factors included gathering information using several questions about whether the household couldn’t afford enough food or eat balanced meals that include fish or meat etc. These food security factors were then assigned points to determine the household hunger scale. The scale used in this study was derived from the Household Hunger Scale from the Food and Nutrition Technical Assistance III Project (FANTA) [48]. The score ranged from 0-8 points, where household with 0–1 points being categorized as “little to no hunger in household”, 2–4 points being categorized as “moderate hunger in household”, and 5–8 points being categorized as “severe hunger in household”.

### 2.6. Statistical analysis

Descriptive statistics of children’s baseline characteristics were computed. The mean and variance of change in the LAZ, WAZ, and WLZ, respectively, relative to the baseline value as well as the prevalence of stunting, wasting, and underweight at each visit were computed and visually displayed for the full cohort and by each subgroup of risk factors in the exploratory analysis. Diarrhea was defined as three or more loose or liquid stools per day, and a new diarrhea episode was defined as onset of diarrhea after three consecutive diarrhea-free days. Spaghetti plots were used to visualize individuals’ trajectories of change in anthropometry z scores and MUAC, and lasagna plots were used to reflect each child’s change in their status of stunting, wasting, underweight, and malnutrition over time. To compare the risk of malnutrition at follow-up between children who were malnourished and those who were not malnourished at enrollment, we constructed a 2 × 2 contingency table based on malnutrition status at enrollment and at day 90. We used Fisher’s exact test to assess differences in proportions and calculated risk ratios with corresponding 95% confidence intervals.

Bivariate and multivariate linear mixed-effect models were conducted to estimate the average change in anthropometry z scores over follow-up and 95% CIs associated with the risk factors of interest. In the bivariate models, the mean model consisted of a linear term of time and potential risk factors (one at a time), with or without an interaction term between them. Variable selection for multivariable models was guided primarily by a priori conceptual frameworks and biological plausibility rather than reliance on p-value thresholds. Key covariates, including age, sex, baseline nutritional status, maternal education, household income, and sanitation and hygiene characteristics, were retained to minimize residual confounding regardless of statistical significance. Given the relatively small sample size, automated or threshold-based variable selection approaches were avoided to reduce the risk of overfitting. In building the multivariable model, we considered potential confounders based on conceptual frameworks by WHO/UNICEF and prior literature. These included child age, sex, baseline anthropometric status, maternal education, household income, and household sanitation and hygiene characteristics. Variables were retained in the final model if they remained statistically significant (p < 0.05). This approach allowed us to adjust for important structural and behavioral factors potentially influencing child growth. Variance Inflation Factor (VIF) was used to check potential multicollinearity. Regarding the random-effects model, a random intercept-only model, a random-slope-only model, and a random intercept and random slope model were explored sequentially for each potential risk factor, using Maximum Likelihood (ML) for model comparison and using Restricted Maximum Likelihood (REML) for coefficient estimates report. Likelihood ratio test (LRT) was used to determine whether an interaction term of fixed effects was included. The selection of random effects models was based on the Akaike information criterion (AIC) and the Bayesian information criterion (BIC), leading to the random intercept and random slope models appearing to be the best-fitting model for all risk factors. Cluster-robust variance estimation was used to obtain the 95% robust CIs of the coefficient estimates.

Although total diarrhea duration over the follow-up period was analyzed to assess its impact on growth outcomes, no significant associations were found; therefore, these results are not reported, and the following results focus on the index case of diarrhea.

The significance level was set to 0.05 for all tests. Data management and analyses were conducted in R (version 4.3.1, 2023-06-16).

## 3. Results

### 3.1. Baseline characteristics of the participants

A total of 138 children under 24 months of age with watery diarrhea, seeking care at Kumudini Hospital in rural Bangladesh, were included in the analysis. Anthropometric measurements were taken at baseline and included five metrics. The median age at baseline was 9.1 months (IQR: 6.0-14.8), and 60.9% of the children were male (Table 1). More than half of the children (55.8%) came from families earning below 20,000 Taka (167.34 USD) per month. In this population, 68.8% of the household heads had an education level of either illiterate or primary, while 52.9% of mothers had completed secondary or higher education. According to the Household Hunger Scale, 5.1% of the population experienced moderate hunger, while none reported severe hunger.

**Table 1:** Baseline characteristics of participants.

	N = 138
**Clinical Outcomes**	
**Severity of diarrhea based on the Modified Vesikari Severity Score**	
Mild (≤ 4)	66 (47.8%)
Moderate-to-Severe (≥ 5)	72 (52.2%)
**Number of loose or liquid stools in the last 24 hours before the presentation**	
≤ 6	43 (31.2%)
7 – 9	48 (34.8%)
≥ 10	47 (34.0%)
**Duration of Diarrhea from Onset (Days)**	
Median [Min, Max]	4.63 [0.0833, 20.6]
**Total Duration of Diarrhea: Index + Future Diarrhea Cases (Days)**	
1 – 3	38 (27.5%)
4 – 5	39 (28.3%)
6 – 9	19 (13.8%)
≥ 10	42 (30.4%)
**Children with future diarrhea cases**	
None	108 (78.3%)
At least one	30 (21.7%)
**Vomiting in the last 48 hours**	
No	43 (31.2%)
Yes	95 (68.8%)
**Fever since diarrhea onset**	
No	84 (60.9%)
Yes	54 (39.1%)
**Dehydration**	
No	122 (88.4%)
Some	15 (10.9%)
Severe	1 (0.7%)
**Hospitalization**	
No	109 (79.0%)
Yes	29 (21.0%)
**Anorexia**	
No	64 (46.4%)
Yes	74 (53.6%)
**Sociodemographic Characteristics**	
**Age (in months)**	
1-6	48 (34.8%)
7-12	47 (34.1%)
13-24	43 (31.2%)
**Sex**	
Male	84 (60.9%)
Female	54 (39.1%)
**Monthly household income (Taka)**	
Below 20,000	77 (55.8%)
20,000 and above	61 (44.2%)
**Household Ratio (# of people living in household/# of bedrooms in household)**	
1-3	94 (68.1%)
3-4	30 (21.7%)
4-5	14 (10.1%)
**Sanitation & Hygiene**	
**Handwashing before preparing food**	
No	27 (19.6%)
Consistently	51 (37.0%)
Sometimes	58 (42.0%)
Does not apply	2 (1.4%)
**Handwashing before feeding children**	
No	14 (10.1%)
Consistently	78 (56.5%)
Sometimes	46 (33.3%)
**Toilet facility**	
Unimproved	32 (23.2%)
Improved	106 (76.8%)
**Disposal of child’s stool**	
Toilet or latrine	44 (31.9%)
Buried	55 (39.9%)
Thrown into the garbage	4 (2.9%)
Put/rinsed into drain/ditch/pond/river	35 (25.4%)
**Type of handwashing facility in the house**	
Tap or faucet	73 (52.9%)
Basin or bucket	1 (0.7%)
Tube well	64 (46.4%)
**How drinking water is stored**	
No storage of water	29 (21.0%)
Closed vessels	86 (62.3%)
Open vessels	23 (16.7%)
**Treatment of drinking water**	
Not treated	130 (94.2%)
Treated (Boiled or Filtered)	8 (5.8%)
**Parental knowledge on the prevention of diarrhea**	
≤ 2 correct answers of 8	81 (58.7%)
≥ 3 correct answers of 8	57 (41.3%)
**Own any animal**	
No	25 (18.1%)
Yes	113 (81.9%)
**Food Security (in the last 6 months)**	
**Household Hunger Scale**	
Little to no hunger in household	131 (94.9%)
Moderate hunger in household	7 (5.1%)

Based on the Modified Vesikari Severity score, 52.2% of children had moderate-to-severe diarrhea, and 34.0% experienced more than ten loose stools within 24 hours of hospital presentation. The median duration of diarrhea from enrollment was 4.63 days [0.08, 20.6], and only 10.9% had signs of any dehydration. Vomiting within 48 hours of hospital presentation was reported in 68.8% of cases, fever was present in 39.1%, and 53.6% had anorexia. Approximately 21.0% of children were hospitalized due to their diarrhea episode.

Sanitation and hygiene practices varied among participants. Over half of the households (52.9%) had tap or faucet facilities for handwashing, while 46.4% relied on tube wells. Improved toilet facilities were present in 76.8% of households, while 23.2% had unimproved facilities. Handwashing practices before preparing food were inconsistent, with 37.0% of caregivers reporting consistent handwashing, 42.0% reporting intermittent handwashing, and 19.6% reporting no handwashing. Similarly, 56.5% of caregivers reported consistent handwashing before feeding children, while 33.3% reported intermittent handwashing, and 10.1% reported no handwashing. Drinking water storage practices also varied, with 62.3% of households storing water in closed vessels, 16.7% in open vessels, and 21.0% reporting no storage of water.

### 3.2. Growth patterns after diarrhea and prevalence of undernutrition over time

The population-average change in length-for-age z-score (LAZ) showed a linear decrease over the three-month follow-up period (Fig 1A), while weight-for-age z-score (WAZ), weight-for-length z-score (WLZ), and mid-upper arm circumference (MUAC) exhibited a linear increase (Fig 1B, 1C, and 1D). There was notable individual variation in the growth trajectories. By the end of the 90-day follow-up, the average changes in z-scores were -0.260 (SD: 0.408) for LAZ, 0.100 (SD: 0.484) for WAZ, 0.249 (SD: 0.725) for WLZ, and MUAC increased by an average of 0.672 cm (SD: 0.476).

**Fig 1:**
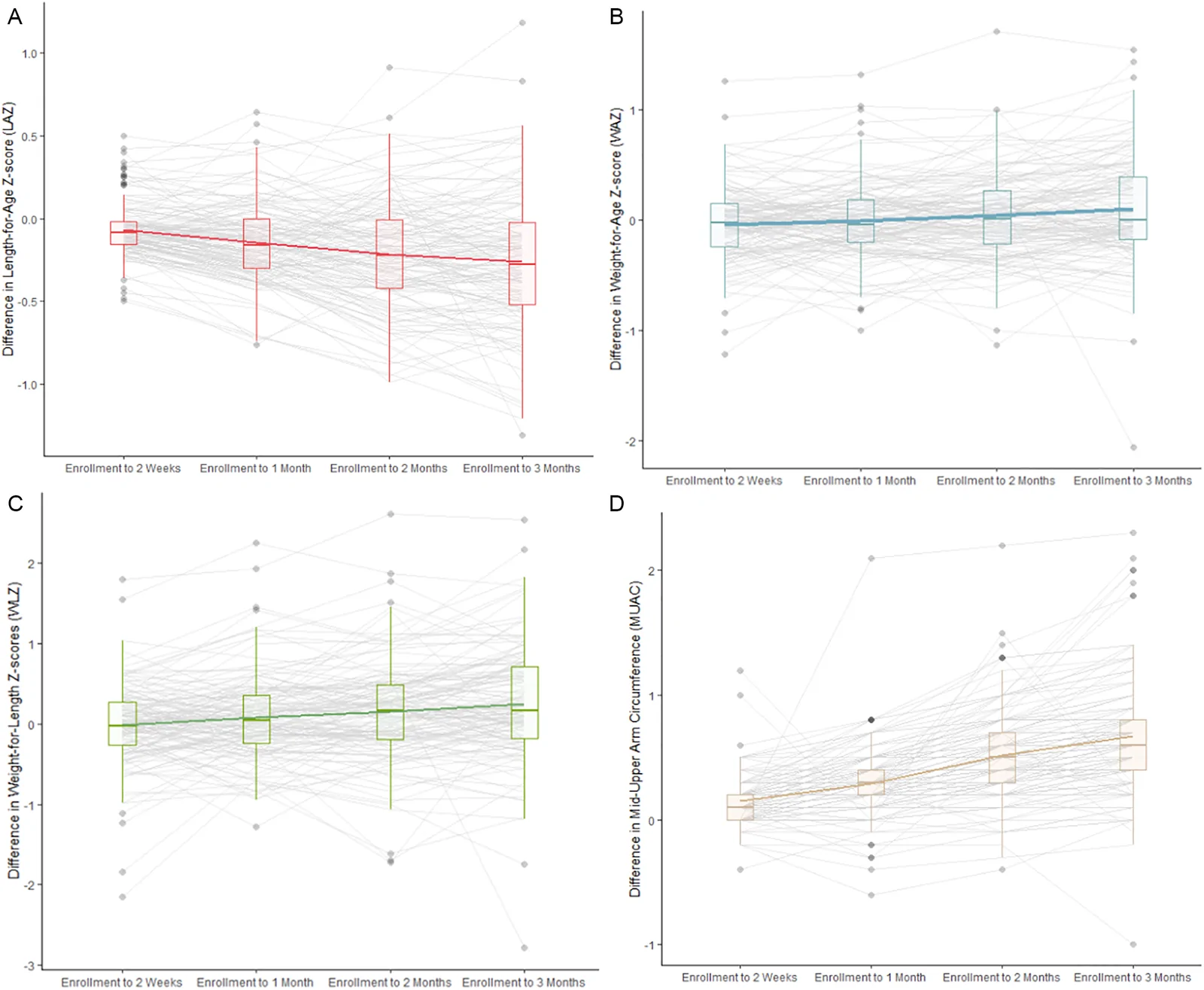
The population average change in Length-for-Age Z-score (a), Weight-for-Age Z-score (b), Weight-for-Length Z-score (c), and Mid-Upper Arm Circumference (MUAC) over the three-month follow-up period with individual growth trajectories.

Although the growth status changed at the individual level for some children, overall, there were no significant changes in the prevalence of stunting, wasting, or undernutrition, as determined by LAZ, WAZ, WLZ, and MUAC scores, over the observation period (Fig 2a-2d). At enrollment, 12 children (8.7%) were stunted, six (4.3%) were wasted, 13 (9.4%) were underweight, and eight (5.8%) had a MUAC ≤12.5 cm, indicating undernutrition. By day 90, four of the stunted children, two of the wasted children, and three of the underweight children had recovered, while the remaining children in each group continued to show deficits. All eight children with low MUAC at enrollment had recovered by day 90. At follow-up, 12 children were stunted—eight who were stunted at baseline and four newly stunted. Similarly, 12 children were underweight at day 90, including 10 who were underweight at enrollment and two new cases. Seven children were wasted at follow-up; four were persistently wasted, and three became newly wasted.

**Fig 2:**
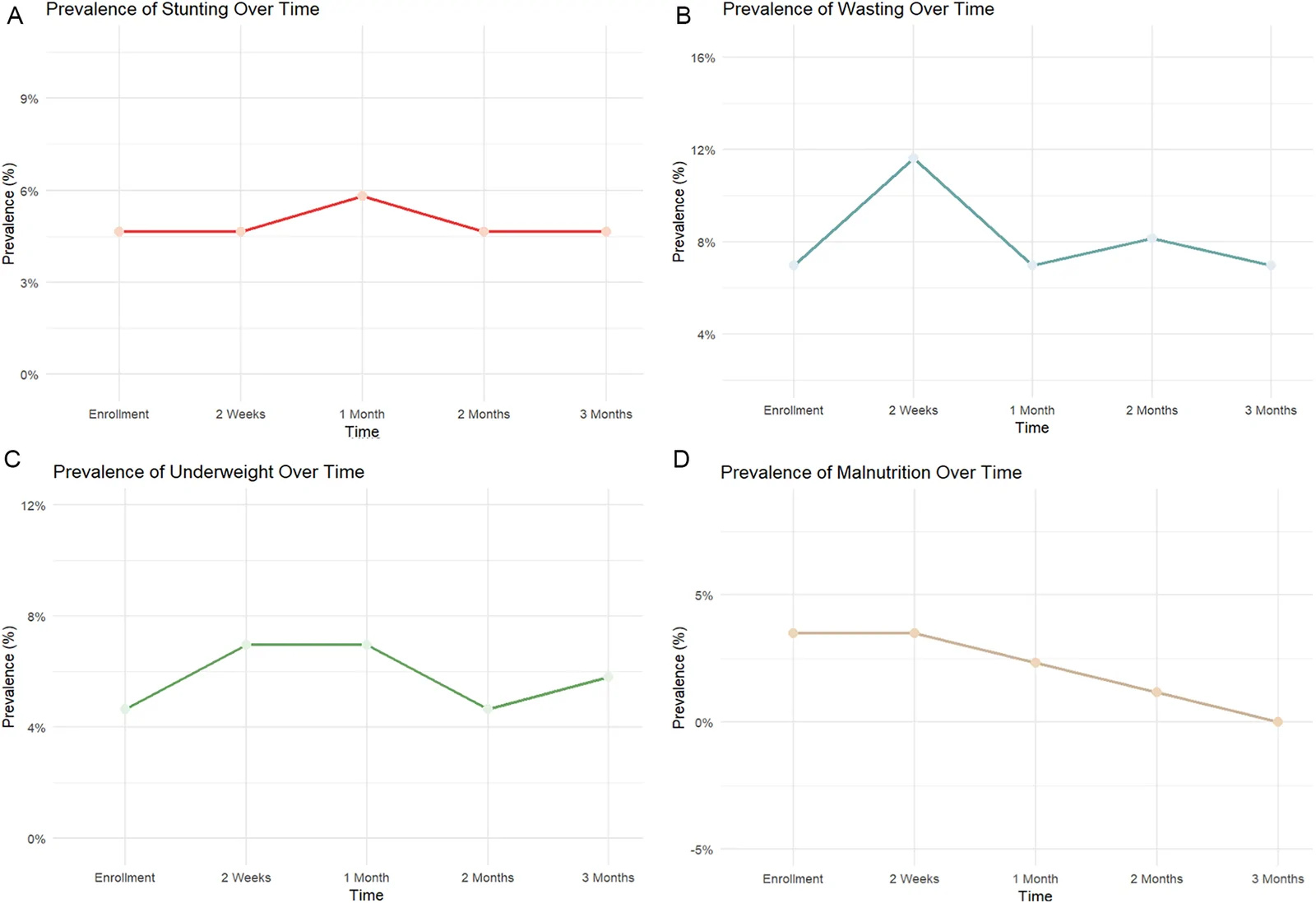
Prevalence of stunting (a), wasting (b), underweight (c), and malnutrition (d) in the study population over the three-month follow-up period post enrollment.

Children who were malnourished at enrollment were significantly more likely to remain malnourished at follow-up than those who were well-nourished at baseline. Of the 25 children who met at least one malnutrition criterion (stunted, wasted, or underweight) at enrollment, 18 (72%) remained malnourished at day 90. In contrast, only 6 of the 113 children (5.3%) who were not malnourished at enrollment developed a new nutritional deficit by follow-up. This corresponds to a risk ratio of 3.8 (p < 0.001, 95% CI: 1.80, 6.35), indicating a significantly higher risk of persistent or incident malnutrition among children who were malnourished at baseline.

### 3.3. Factors associated with change in growth parameters

Tables 2 and S1–S4 present the results of bivariate and multivariate linear mixed-effects models assessing the association between changes in WAZ, LAZ, WLZ, and MUAC and sociodemographic, sanitation, hygiene, food security, and clinical risk factors.

**Table 2:** Risk factors associated with change in LAZ, WAZ, WLZ, MUAC during study follow-up among children under 24 months of age with watery diarrhea seeking care at Kumudini Hospital in Bangladesh.

	ΔLAZ		ΔWAZ		ΔWLZ		ΔMUAC	
	Adjusted difference (95% CI)^acd^	p-val^d^	Adjusted difference (95% CI)^acd^	p-val^d^	Adjusted difference (95% CI)^acd^	p-val^d^	Adjusted difference (95% CI)^acd^	p-val^d^
**Clinical Factors**								
**Index Diarrhea Duration (in days)**								
0-3	ref		ref		ref		ref	
3-5	0.006 (-0.077, 0.090)	0.877	-0.098 (-0.238, 0.041)	0.165	–	–	-0.044 (-0.135, 0.047)	0.335
5-7	-0.015 (-0.119, 0.090)	0.776	-0.109 (-0.294, 0.077)	0.244	–	–	-0.034 (-0.147, 0.079)	0.545
≥ 7	**-0.085** **(-0.184, 0.014)**	**0.090**	-0.094 (-0.221, 0.032)	0.141	–	–	-0.070 (-0.161, 0.020)	0.124
**Vomiting within 48 hours**								
No	ref		ref		ref		ref	
Yes	**–**	**–**	0.036 (-0.089, 0.161)	0.565	0.136 (-0.086, 0.357)	0.224	–	–
**Hospitalization at enrollment for diarrhea**								
No	ref		ref		ref		ref	
Yes	**-0.095** **(-0.164, -0.025)**	**0.009**	-0.062 (-0.196, 0.072)	0.357	-0.041 (-0.252, 0.169)	0.695	**-0.090** **(-0.179, -0.0004)**	**0.049**
**Anorexia**								
No	ref		ref		ref		ref	
Yes	–	–	–	–	–	–	-0.020 (-0.096, 0.056)	0.609
**Sociodemographic Factors**								
**Baseline age (months)**								
1-6	ref		ref		ref		ref	
7-12	0.065 (-0.031, 0.160)	0.180	**-0.109** **(-0.239, 0.020)**	**0.096**	-0.181 (-0.405, 0.044)	0.113	-0.031 (-0.096, 0.056)	0.553
13-24	0.067 (-0.021, 0.155)	0.134	-0.070 (-0.239, 0.100)	0.415	-0.116 (-0.390, 0.157)	0.398	-0.022 (-0.123, 0.080)	0.669
**Sex**								
Male	ref		ref		ref		ref	
Female	0.037 (-0.030, 0.105)	0.276	-0.014 (-0.117, 0.090)	0.784	-0.055 (-0.222, 0.113)	0.516	0.032 (-0.043, 0.107)	0.401
**Head of household education**								
Illiterate, no education, incomplete primary	ref		ref		ref		ref	
Completed primary, completed secondary	0.033 (-0.044, 0.110)	0.394	–	–	-0.086 (-0.288, 0.116)	0.397	**0.094** **(0.006, 0.182)**	**0.037**
Completed higher education	-0.050 (-0.150, 0.050)	0.313	–	–	-0.021 (-0.263, 0.221)	0.861	-0.043 (-0.160, 0.074)	0.456
**Mother’s Education**								
Illiterate, no education, incomplete primary	ref		ref		ref		ref	
Completed primary, completed secondary	0.056 (-0.033, 0.144)	0.209	–	–	–	–	-0.029 (-0.137, 0.079)	0.591
Completed higher education	-0.021 (-0.111, 0.069)	0.638	**–**	**–**	–	–	-0.047 (-0.168, 0.074)	0.442
**Monthly household income (Taka)**								
≤ 20,000	ref		ref		ref		ref	
> 20,000	0.015 (-0.055, 0.086)	0.665	-0.024 (-0.146, 0.097)	0.691	-0.092 (-0.288, 0.105)	0.356	-0.017 (-0.110, 0.074)	0.718
**Owns any animals**								
No	ref		ref		ref		ref	
Yes	–	–	–	–	–	–	-0.040 (-0.142, 0.062)	0.427
**Parental knowledge on the prevention of diarrhea**								
≤ 2 correct answers of 8	ref		ref		ref		ref	
≥ 3 correct answers of 8	**0.069** **(-0.007, 0.150)**	**0.073**	–	–	-0.010 (-0.194, 0.173)	0.911	–	–
**Household Ratio (# of people living in the household/# of bedrooms in the household)**								
1–3 people per bedroom	ref		ref		ref		ref	
3–4 people per bedroom	-0.020 (-0.111, 0.071)	0.662	0.001 (-0.172, 0.174)	0.991	0.073 (-0.185, 0.331)	0.570	-0.057 (-0.139, 0.025)	0.165
4–5 people per bedroom	0.022 (-0.108, 0.152)	0.726	-0.049	0.548	-0.134 (-0.430, 0.163)	0.358	-0.039 (-0.148, 0.070)	0.466
**Household Hunger Scale**								
Little-to-no hunger in the household	ref		ref		ref		ref	
Moderate hunger in the household	0.085 (-0.098, 0.268)	0.320	**-0.224** **(-0.402, -0.045)**	**0.021**	**-0.429** **(-0.790, -0.069)**	**0.025**	0.085 (-0.149, 0.318)	0.430
**Sanitation and Hygiene Factors**								
**Handwashing with soap before feeding children**								
No	ref		ref		ref		ref	
Sometimes or consistently	0.096 (-0.048, 0.240)	0.175	–	–	–	–	–	–
**Handwashing after using the toilet**								
No	ref		ref		ref		ref	
Sometimes or consistently	-0.137 (-0.340, 0.065)	0.138	–	–	–	–	–	–
**Toilet Facility**								
Unimproved	ref		ref		ref		ref	
Improved	–	–	-0.007 (-0.155, 0.142)	0.929	0.026 (-0.231, 0.283)	0.839	–	–
**Disposal of child’s stool**								
Toilet or latrine	ref		ref		ref		ref	
Buried	–		–		**-0.182** **(-0.387, 0.023)**	**0.081**	-0.010 (-0.104, 0.084)	0.835
Thrown into the garbage	–		–		-0.011 (-0.443, 0.420)	0.945	0.002 (-0.321, 0.326)	0.985
Put/rinsed into drain/ditch/pond/river	–		–		-0.040 (-0.292, 0.211)	0.750	-0.009 (-0.100, 0.079)	0.844
**Type of handwashing facility in the household**								
Tap or faucet	ref		ref		ref		ref	
Basin or bucket	0.052 (-0.187, 0.29)	0.557	**-0.566** **(-1.311, 0.179)**	**0.076**	**-0.783** **(-1.72, 0.154)**	**0.068**	-0.208 (-0.520, 0.104)	0.144
Tube Well	-0.026 (-0.097, 0.044)	0.463	0.019 (-0.096, 0.128)	0.775	0.089 (0.079, 0.256)	0.293	-0.008 (-0.087, 0.071)	0.845
**How drinking water is stored**								
No storage of water	ref		ref		ref		ref	
Closed Containers	0.034 (-0.042, 0.110)	0.376	–	–	**–**	**–**	–	–
Open Containers	0.062 (-0.041, 0.166)	0.230	–	–	–	–	–	–
**Treatment of drinking water**								
Not treated	ref		ref		ref		ref	
Treated (Boiled/Filter)	-0.011 (-0.190, 0.168)	0.899	0.087 (-0.102, 0.276)	0.331	0.102 (-0.244, 0.449)	0.527	–	–

^a^Estimates of the best-fitting linear mixed-effects models, i.e., the random intercept and random slope models, based on AIC and BIC. Model comparison was based on Maximum Likelihood estimated models, while the reported coefficients and 95% confidence intervals (CIs) of the final model was obtained from Restricted Maximum Likelihood estimation.

^b^An interaction term of the risk factor with time was included only when a p-Value of <0.05 in the Likelihood Ratio Test comparing the full model with the interaction term and the nested model without the interaction term.

^c^Risk factors or their interaction terms with the time that had a p-Value of <0.20 in the bivariate analysis were included in the multivariate model.

^d^95% CIs of the risk differences and the p-Values were based on cluster-robust variance estimation with CR2 small sample correction.

Abbreviations: MUAC – mid-upper arm circumference; CI – confidence interval; p-val – p-Value; ref – reference.

Fixed effects, statistically significant results (p < 0.05) and borderline significant results (p < 0.10) are bold.

#### 3.3.1. Clinical factors.

In bivariate models, clinical conditions such as diarrhea duration, vomiting, and hospitalization were associated with significant negative changes in anthropometric outcomes (S1a Table). Children whose diarrheal disease required hospitalization at enrollment experienced a notable decrease in LAZ by 0.095 (p = 0.009, 95% CI: -0.164, -0.025). Children who had 7 days or more of diarrhea had a decrease in LAZ score of 0.085 (p = 0.090, 95% CI: -0.184, 0.014) at follow-up measurements than children who had diarrhea duration for zero to three days. Children who were hospitalized at enrollment for their diarrheal illness experienced lower MUAC scores at follow-up by 0.098 (p = 0.030, 95% CI: -0.185, -0.010).

#### 3.3.2. Sociodemographic factors.

In the bivariate analyses, age emerged as a significant demographic factor impacting nutritional outcomes (S2 Table). Children aged 7–12 months had LAZ scores that were 0.161 higher (p = 0.008, 95% CI: 0.043, 0.278) compared to those younger than 6 months, while children aged 13–24 months showed a 0.113 increase in LAZ (p = 0.025, 95% CI: 0.015, 0.211) compared to those younger than 6 months at enrollment. However, age also modified the effect of time on weight and length measures; older children demonstrated a greater decline in WAZ, with children aged 7–12 months experiencing a 0.068 decrease (p = 0.022, 95% CI: -0.125, -0.010) and those aged 13–24 months showing a 0.096 decline (p < 0.001, 95% CI: -0.151, -0.042) relative to younger children. Similar trends were observed in WLZ, where older children had a greater decline over time compared to children under 6 months.

In the bivariate model, children in households with a higher occupancy ratio (3–4 people per bedroom) exhibited lower LAZ scores by 0.129 (p = 0.025, 95% CI: -0.241, -0.017) compared to those in less crowded homes. Higher income and maternal education levels were generally associated with improved scores across WAZ, WLZ, although specific values were not statistically significant after adjustment.

In the multivariate, adjusted model, children between the ages of 7 and 12 months experienced lower WAZ and WLZ scores when compared to children 1–6 months old. Their WAZ scores declined by 0.129 (p = 0.057, 95% CI: -0.262, 0.004) and their WLZ scores decreased by 0.242 (p = 0.049, 95% CI: -0.483, -0.0008) by the 90-day follow-up. Parental knowledge on the prevention of diarrhea impacted LAZ scores positively. Parents who had more knowledge in diarrhea prevention methods had LAZ scores that were greater by 0.069 (p = 0.073, 95% CI: -0.007, 0.150).

#### 3.3.3. Sanitation and hygiene factors.

Sanitation and hygiene practices played a significant role in influencing growth outcomes (S3 Table). Handwashing frequency and the type of handwashing facility were associated with notable changes in anthropometric measures. For example, children whose guardians practiced handwashing before feeding them had LAZ scores 0.155 higher (p = 0.009, 95% CI: 0.045, 0.266) than those who did not. However, the use of a basin or bucket for handwashing was associated with a decline in scores, with children using these facilities having lower WAZ scores by 0.618 (p < 0.001, 95% CI: -0.713, -0.522), lower LAZ scores by 0.078 (p = 0.017, 95% CI: -0.142, -0.014), reduced WLZ (decrease of 0.740, p < 0.001, 95% CI: -0.889, -0.590), and MUAC (decrease of 0.300, p < 0.001, 95% CI: -0.359, -0.241). After adjustment, in the multivariate model, the type of handwashing facility did not impact the LAZ and MUAC measurements significantly but had borderline significance in the decrease of WAZ (decrease of 0.566, p = 0.076, 95% CI: -1.311, 0.179) and WLZ (decrease of 0.783, p = 0.068, 95% CI: -1.72, 0.154) measurements.

Access to improved sanitation also contributed positively to outcomes. Children in households with improved toilet facilities exhibited a 0.063 increase in WAZ (p = 0.017, 95% CI: 0.012, 0.115) and a 0.109 increase in WLZ (p = 0.007, 95% CI: 0.030, 0.188) scores over time.

#### 3.3.4. Food security factors.

Food security and access to balanced meals were critical determinants of nutritional outcomes (S4 Table). Limited access to protein-rich foods was associated with lower growth scores. Children from households that struggled to afford balanced meals containing fish or meat over the previous six months had significantly lower LAZ scores at follow-up by 0.132 (p = 0.042, 95% CI: -0.259, -0.005) compared to children with more consistent access to these foods.

The Household Hunger Scale provided further insights, indicating no cases of severe hunger and only a few instances of moderate hunger within the study population. Children from households with moderate hunger demonstrated declines in both WAZ and WLZ. Specifically, moderate hunger was associated with a 0.224 decrease in WAZ (p = 0.021, 95% CI: -0.402, -0.045) and a 0.429 reduction in WLZ (p = 0.025, 95% CI: -0.790, 0.069) at follow-up compared to children from households with little to no hunger. These findings underscore the role of food security in maintaining and improving nutritional outcomes among young children.

## 4. Discussion

This study provides valuable insights into the determinants of post-diarrhea growth outcomes among children under two in rural Bangladesh. Our findings suggest that growth outcomes following diarrheal episodes are associated with a complex interplay of clinical, sociodemographic, sanitation, hygiene, and food security factors.

Children who were already malnourished at enrollment represent a particularly vulnerable subgroup that needs to be identified as quickly as possible as these children are at higher risk of persistent growth faltering and require targeted follow-up care to break the vicious cycle of undernutrition and recurrent infections. Our findings indicate that baseline malnutrition strongly predicts persistent or recurrent malnutrition. The vulnerability of this subgroup warrants the need of integrated interventions- combining nutritional support, healthcare access, and caregiver education- to address their specific needs. The strong association between baseline malnutrition and subsequent malnutrition highlights a critical opportunity for early intervention. These findings suggest that nutritional support strategies should be initiated at the time of first clinical contact rather than delayed, as children presenting with malnutrition are at substantially higher risk of persistent growth deficits.

The clinical burden of prolonged diarrhea emerged as a significant factor in growth declines, aligning with previous studies showing the detrimental impact of extended diarrheal episodes on nutritional recovery [22–24,38,49–52]. Children with longer diarrhea durations exhibited lower growth indicators, suggesting that the duration and severity of symptoms like vomiting, dehydration, fever, and anorexia might exacerbate malnutrition. This indicates the importance of sustained clinical support for children recovering from extended diarrheal periods to help mitigate the compounding effects of prolonged symptoms on growth outcomes. Effective post-discharge care may include support and follow-up for symptom management and nutritional supplementation, which could enhance recovery and resilience [39,40].

Younger children demonstrated more favorable growth responses in comparison to older children at enrollment, showing that age played an essential role in post-diarrhea growth outcomes. This may reflect the cumulative health burden carried by older children who have likely experienced repeated infections, weakening their resilience over time [24,26,41,42]. Additionally, household environment and sociodemographic factors, including family size, maternal education, and household income, were associated with variations in growth measures. These findings underscore that economic resources and educational background influence caregivers’ ability to maintain conditions that promote child growth. Socioeconomic interventions that enhance maternal knowledge of child health and nutrition could therefore help alleviate the cycle of infection and malnutrition in early childhood [43,44,53,54].

Sanitation and hygiene factors were also significantly associated with growth outcomes. Consistent handwashing practices among caregivers correlated with better growth outcomes, while shared or inadequate sanitation facilities were linked to an increased risk of growth faltering. The use of basins or buckets for handwashing was associated with poorer growth outcomes, suggesting that the type of handwashing facility may play a role in hygiene effectiveness [55,56]. Hygiene interventions were previously shown to significantly reduce diarrhea prevalence and improve child growth outcomes, further underscoring the importance of proper handwashing practices [57–59]. These findings reinforce the role of environmental sanitation in child health, aligning with literature that associates poor sanitation and hygiene with increased pathogen exposure and environmental enteric dysfunction [26,35,45,46,58–61]. Initiatives to improve community-level sanitation infrastructure, alongside education to reinforce proper hand hygiene, could substantially reduce the transmission of diarrheal pathogens and their impact on child growth [47,57,59]. However, the effectiveness of WASH (Water, Sanitation, and Hygiene) interventions has shown mixed results in some studies, with varying impacts on child growth and diarrhea prevalence [10,31]. This underscores the need for context-specific approaches that address local barriers and challenges to WASH implementation and adherence.

Furthermore, food security emerged as a critical factor, with households facing moderate hunger or limited access to protein-rich foods demonstrating poorer growth outcomes. Consistent with existing research, these findings highlight the importance of adequate dietary intake for growth recovery following infection and echoes the WASH Benefits study conclusion that nutrition interventions had stronger growth effects than WASH alone [3,62–64]. Protein and other essential nutrients play an important role in immune function and growth, emphasizing the need for nutritional programs that prioritize food security. Programs that provide regular monitoring and supplementation, such as ready-to-use therapeutic foods (RUTF), have shown promise in improving growth outcomes in similar settings [31]. Integrating the Household Hunger Scale into routine assessments could help identify at-risk families and provide targeted support, such as RUTF, thereby enhancing child resilience against future growth challenges [65,66].

Some findings in this study were interesting and warrant further discussion. For instance, the continuous decline in length-for-age z-scores (LAZ) over the follow-up period contrasts with previous studies that have reported catch-up growth following diarrheal episodes [67,68]. Although growth velocity has been proposed as a sensitive indicator of post-illness recovery, our analysis focused on changes in anthropometric z-scores across discrete follow-up time points. Future studies may benefit from incorporating velocity-based measures to capture short-term growth dynamics better. A recent study by Brander et al. also found that linear growth faltering was strongly associated with diarrheal episodes, particularly in children with moderate-to-severe diarrhea, supporting our findings [22]. Additionally, the negative association between higher maternal education and LAZ scores was unexpected, as maternal education is typically associated with improved child health outcomes [69,70]. This finding may reflect confounding factors, such as working mothers having less time for childcare or differences in feeding practices and warrants further investigation.

Although the total duration of subsequent diarrheal episodes was evaluated, no statistically significant associations with growth outcomes were observed in this analysis. The focus on the index diarrheal episode was therefore retained to maintain consistency to examine early post-illness recovery trajectories. However, the absence of observed associations with cumulative duration may reflect limited statistical power or measurement constraints rather than the absence of a true biological relationship. Future studies with larger sample sizes and longer follow-up periods are needed to better evaluate the potential impact of recurrent or prolonged diarrheal illness on child growth.

This study has several strengths. The longitudinal design allowed for the examination of growth trajectories over time, providing insights into the dynamic nature of child growth following diarrheal episodes. The use of standardized anthropometric measurements and validated tools, such as the Modified Vesikari Severity Score and the Household Hunger Scale, enhances the reliability and comparability of the findings [38,48,65]. Additionally, the inclusion of a wide range of clinical, sociodemographic, and environmental factors provides a comprehensive understanding of the determinants of post-diarrhea growth outcomes. This study also contributes to the growing body of literature on the interplay between diarrhea, nutrition, and child growth in low-resource settings, offering valuable evidence for the design of targeted interventions [71,72].

This study has limitations. First, the relatively small sample size may have limited statistical power to detect modest associations and increased the uncertainty of estimates. As such, the findings should be interpreted as exploratory and hypothesis-generating rather than definitive. Second, the single-site design may limit generalizability to other populations and studies. Third, the exclusion of children with severe acute malnutrition and restriction to watery diarrhea cases may introduce selection bias toward relatively healthier children. Finally, given the observational nature of the study, the associations identified should not be interpreted as causal.

## 5. Conclusion

In conclusion, this study highlights the multifactorial nature of child growth and the critical role of age, household practices, environmental factors, and clinical symptoms in influencing nutritional outcomes after diarrhea incidence. Interventions that focus on improving hygiene, sanitation, and access to healthcare, alongside age-specific nutritional education, are essential for supporting child growth and development. By addressing these determinants, public health initiatives can effectively combat malnutrition and enhance the overall well-being of children.

### 5.1. Importance

Children under two years of age in low and middle-income countries are highly vulnerable to undernutrition, especially following diarrheal illness. This study shows that growth recovery after diarrhea is not guaranteed and is shaped by multiple factors, including the child’s age, the severity of illness, household sanitation and hygiene practices, and food security. Children who were already malnourished at the time of illness were much more likely to remain so months later. Poor hygiene practices and limited access to protein-rich foods further reduced the likelihood of recovery. These findings are critical because they highlight the need for targeted follow-up and integrated interventions to support children after diarrhea. By identifying which children are at highest risk and which environmental and clinical conditions hinder growth, this study provides essential evidence to guide strategies for improving child nutrition and health in similar low-resource settings.

## Supporting information

S1 Table Clinical risk factors associated with the crude change in LAZ, WAZ, WLZ, and MUAC during study follow-up among children under 24 months of age with watery diarrhea seeking care at Kumudini Hospital in Bangladesh. (DOCX)

S2 Table Sociodemographic factors associated with the crude change in LAZ, WAZ, WLZ, and MUAC during study follow-up among children under 24 months of age with watery diarrhea seeking care at Kumudini Hospital in Bangladesh. (DOCX)

S3 Table Sanitation and Hygiene risk factors associated with the crude change in LAZ, WAZ, WLZ, and MUAC during study follow up among children under 24 months of age with watery diarrhea seeking care at Kumudini Hospital in Bangladesh. (DOCX)

S4 Table Food Security risk factors associated with the crude change in LAZ, WAZ, WLZ, and MUAC during study follow-up among children under 24 months of age with watery diarrhea seeking care at Kumudini Hospital in Bangladesh. (DOCX)

S1 Fig Directed acyclic graph (DAG) illustrating the hypothesized causal relationships between sociodemographic characteristics, environmental exposures, and diarrheal illness in relation to child growth. Confounders are shown in blue, exposures in green, mediators in orange, and the outcome in red. Arrows represent assumed causal pathways, including both direct and indirect effects mediated through diarrheal severity and is sequelae. Abbreviations: Baseline, baseline nutrition; MatEdu, maternal education; Sanit, sanitation; Hyg, hygiene; FoodSec, food security; Severity, diarrhea severity; Duration, diarrhea duration; Hosp, hospitalization.
(TIFF)

## References

[1] WHO. Global Targets 2025: To improve maternal, infant and young child nutrition. Published 2012. [cited 2024 Nov 19]. Available from: https://www.who.int/teams/nutrition-and-food-safety/global-targets-2025

[2] United Nations. Sustainable Development Goal 2: End hunger, achieve food security and improved nutrition and promote sustainable agriculture. 2015. [cited 2023 Nov 19]. Available from: https://sdgs.un.org/goals/goal2#targets_and_indicators

[3] Victora CG, Christian P, Vidaletti LP, Gatica-Domínguez G, Menon P, Black RE. Revisiting maternal and child undernutrition in low-income and middle-income countries: Variable progress towards an unfinished agenda. Lancet. 2021;397(10282):1388–99. doi: 10.1016/S0140-6736(21)00394-9 33691094 PMC7613170

[4] UNICEF, WHO, World Bank Group. Levels and Trends in Child Malnutrition: UNICEF/WHO/World Bank Group Joint Child Malnutrition Estimates: Key Findings of the 2023 Edition. 2023. [cited 2023 Nov 19]. Available from: https://www.who.int/publications/i/item/9789240073791

[5] World Health Organization. Underweight prevalence among children under 5 years of age (%). 2022. [cited 2023 Feb 28]. Available from: https://www.who.int/data/gho/data/indicators/indicator-details/GHO/gho-jme-underweight-prevalence

[6] Black RE, Victora CG, Walker SP, Bhutta ZA, Christian P, de Onis M, et al. Maternal and child undernutrition and overweight in low-income and middle-income countries. Lancet. 2013;382(9890):427–51. doi: 10.1016/S0140-6736(13)60937-X 23746772

[7] De Sanctis V, Soliman A, Alaaraj N, Ahmed S, Alyafei F, Hamed N. Early and long-term consequences of nutritional stunting: from childhood to adulthood. Acta Biomed. 2021;92(1):e2021168. doi: 10.23750/abm.v92i1.11346 33682846 PMC7975963

[8] World Health Organization. Malnutrition: fact sheet. 2021. [cited 2023 Feb 26]. Available from: https://www.who.int/news-room/fact-sheets/detail/malnutrition

[9] Mertens A, Benjamin-Chung J, Colford JM Jr, Hubbard AE, van der Laan MJ, Coyle J, et al. Child wasting and concurrent stunting in low- and middle-income countries. Nature. 2023;621(7979):558–67. doi: 10.1038/s41586-023-06480-z 37704720 PMC10511327

[10] Luby SP, Halder AK, Huda T, Unicomb L, Johnston RB. The effect of handwashing at recommended times with water alone and with soap on child diarrhea in rural Bangladesh: An observational study. PLoS Med. 2011;8(6):e1001052. doi: 10.1371/journal.pmed.1001052 21738452 PMC3125291

[11] The World Bank. Prevalence of underweight, weight for age (% of children under 5) - Bangladesh. [cited 2023 Mar 2]. Available from: https://data.worldbank.org/indicator/SH.STA.MALN.ZS?locations=BD

[12] The World Bank. Prevalence of stunting, height for age (% of children under 5) - Bangladesh. [cited 2023 Mar 2]. Available from: https://data.worldbank.org/indicator/SH.STA.STNT.ZS?locations=BD

[13] The World Bank. Prevalence of wasting, weight for height (% of children under 5) - Bangladesh. [cited 2023 Mar 2]. Available from: https://data.worldbank.org/indicator/SH.STA.WAST.ZS?locations=BD-1W

[14] Bangladesh Bureau of Statistics (BBS), UNICEF Bangladesh. Progotir Pathey, Bangladesh Multiple Indicator Cluster Survey 2019, Survey Findings Report. 2019. [cited 2023 Mar 2]. Available from: https://www.unicef.org/bangladesh/media/3281/file/Bangladesh%202019%20MICS%20Report_English.pdf

[15] Chowdhury MRK, Rahman MS, Khan MMH, Mondal MNI, Rahman MM, Billah B. Risk factors for child malnutrition in Bangladesh: A multilevel analysis of a nationwide population-based survey. J Pediatr. 2016;172:194-201.e1. doi: 10.1016/j.jpeds.2016.01.023 26858194

[16] UNICEF. UNICEF Conceptual Framework on Maternal and Child Nutrition. 2021. [cited 2023 Mar 4]. Available from: https://www.unicef.org/media/113291/file/UNICEF%20Conceptual%20Framework.pdf

[17] Wirth JP, Rohner F, Petry N, Onyango AW, Matji J, Bailes A, et al. Assessment of the WHO stunting framework using Ethiopia as a case study. Matern Child Nutr. 2017;13(2):e12310. doi: 10.1111/mcn.12310 27126511 PMC6865882

[18] Danaei G, Andrews KG, Sudfeld CR, Fink G, McCoy DC, Peet E, et al. Risk factors for childhood stunting in 137 developing countries: a comparative risk assessment analysis at global, regional, and country levels. PLoS Med. 2016;13(11):e1002164. doi: 10.1371/journal.pmed.1002164 27802277 PMC5089547

[19] Katoch OR. Determinants of malnutrition among children: A systematic review. Nutrition. 2022;96:111565. doi: 10.1016/j.nut.2021.111565 35066367

[20] Siddiqa M, Zubair A, Kamal A, Ijaz M, Abushal T. Prevalence and associated factors of stunting, wasting and underweight of children below five using quintile regression analysis (PDHS 2017-2018). Sci Rep. 2022;12(1):20326. doi: 10.1038/s41598-022-24063-2 36434025 PMC9700674

[21] Soboksa NE, Gari SR, Hailu AB, Mengistie Alemu B. Childhood malnutrition and the association with diarrhea, water supply, sanitation, and hygiene practices in Kersa and Omo Nada Districts of Jimma Zone, Ethiopia. Environ Health Insights. 2021;15. doi: 10.1177/1178630221999635 33746513 PMC7940723

[22] Brander RL, Pavlinac PB, Walson JL, John-Stewart GC, Weaver MR, Faruque ASG, et al. Determinants of linear growth faltering among children with moderate-to-severe diarrhea in the Global Enteric Multicenter Study. BMC Med. 2019;17(1):214. doi: 10.1186/s12916-019-1441-3 31767012 PMC6878715

[23] Richard SA, Black RE, Gilman RH, Guerrant RL, Kang G, Lanata CF, et al. Diarrhea in early childhood: short-term association with weight and long-term association with length. Am J Epidemiol. 2013;178(7):1129–38. doi: 10.1093/aje/kwt094 23966558 PMC3783094

[24] Richard SA, Black RE, Gilman RH, Guerrant RL, Kang G, Lanata CF, et al. Diarrhea in early childhood: Short-term association with weight and long-term association with length. Am J Epidemiol. 2013;178(7):1129–38. doi: 10.1093/aje/kwt094 23966558 PMC3783094

[25] Chowdhury MMH, Khandker S, Chowdhury MAH, Hossain MK, Rahman SMM. Factors affecting the incidence of diarrhoeal disease among children under 2 years in remote village area of Bangladesh. Child Care Health Dev. 2026;52(1):e70228. doi: 10.1111/cch.70228 41457791

[26] Hossain MS, Begum SMKN, Rahman MM, Parvez M, Mazumder RN, Sarker SA, et al. Environmental enteric dysfunction and small intestinal histomorphology of stunted children in Bangladesh. PLoS Negl Trop Dis. 2023;17(1):e0010472. doi: 10.1371/journal.pntd.0010472 36656867 PMC9888706

[27] Wolf J, Prüss-Ustün A, Cumming O, Bartram J, Bonjour S, Cairncross S, et al. Assessing the impact of drinking water and sanitation on diarrhoeal disease in low- and middle-income settings: Systematic review and meta-regression. Trop Med Int Health. 2014;19(8):928–42. doi: 10.1111/tmi.12331 24811732

[28] Clasen TF, Alexander KT, Sinclair D, Boisson S, Peletz R, Chang HH, et al. Interventions to improve water quality for preventing diarrhoea. Cochrane Database Syst Rev. 2015;2015(10):CD004794. doi: 10.1002/14651858.CD004794.pub3 26488938 PMC4625648

[29] Luby SP, Rahman M, Arnold BF, Unicomb L, Ashraf S, Winch PJ, et al. Effects of water quality, sanitation, handwashing, and nutritional interventions on diarrhoea and child growth in rural Bangladesh: A cluster randomised controlled trial. Lancet Glob Health. 2018;6(3):e302–15. doi: 10.1016/S2214-109X(17)30490-4 29396217 PMC5809718

[30] Humphrey JH, Mbuya MNN, Ntozini R, Moulton LH, Stoltzfus RJ, Tavengwa NV, et al. Independent and combined effects of improved water, sanitation, and hygiene, and improved complementary feeding, on child stunting and anaemia in rural Zimbabwe: A cluster-randomised trial. Lancet Glob Health. 2019;7(1):e132–47. doi: 10.1016/S2214-109X(18)30374-7 30554749 PMC6293965

[31] Cumming O, Arnold BF, Ban R, Clasen T, Esteves Mills J, Freeman MC, et al. The implications of three major new trials for the effect of water, sanitation and hygiene on childhood diarrhea and stunting: A consensus statement. BMC Med. 2019;17(1):173. doi: 10.1186/s12916-019-1410-x 31462230 PMC6712663

[32] Ahmed T, Mahfuz M, Islam MM, et al. The MAL-ED cohort study in Mirzapur, Bangladesh: Methods and challenges in data collection. PLoS One. 2020;15(7):e0235704. doi: 10.1371/journal.pone.0235704

[33] Checkley W, Buckley G, Gilman RH, Assis AM, Guerrant RL, Morris SS, et al. Multi-country analysis of the effects of diarrhoea on childhood stunting. Int J Epidemiol. 2008;37(4):816–30. doi: 10.1093/ije/dyn099 18567626 PMC2734063

[34] Kosek M, Haque R, Lima A, Babji S, Shrestha S, Qureshi S, et al. Fecal markers of intestinal inflammation and permeability associated with the subsequent acquisition of linear growth deficits in infants. Am J Trop Med Hyg. 2013;88(2):390–6. doi: 10.4269/ajtmh.2012.12-0549 23185075 PMC3583335

[35] Campbell RK, Schulze KJ, Shaikh S, Mehra S, Ali H, Wu L, et al. Biomarkers of environmental enteric dysfunction among children in rural Bangladesh. J Pediatr Gastroenterol Nutr. 2017;65(1):40–6. doi: 10.1097/MPG.0000000000001557 28644348 PMC5492885

[36] World Health Organization. Diarrhoeal disease: fact sheets. 2017. [cited 2023 Mar 7]. Available from: https://www.who.int/news-room/fact-sheets/detail/diarrhoeal-disease

[37] Ferdous F, Das SK, Ahmed S, Farzana FD, Latham JR, Chisti MJ, et al. Severity of diarrhea and malnutrition among under five-year-old children in rural Bangladesh. Am J Trop Med Hyg. 2013;89(2):223–8. doi: 10.4269/ajtmh.12-0743 23817334 PMC3741240

[38] Fuchs C, Sultana T, Ahmed T, Iqbal Hossain M. Factors associated with acute malnutrition among children admitted to a diarrhoea treatment facility in Bangladesh. Int J Pediatr. 2014;2014:267806. doi: 10.1155/2014/267806 24734048 PMC3964844

[39] Chakraborty S, Dash S, Antara NA, Roy BR, Mamun SA, Ali M, et al. The Impact of non-dysentery shigella infection on the growth and health of children over time (INSIGHT)-A prospective case-control study protocol. Microorganisms. 2024;12(8):1677. doi: 10.3390/microorganisms12081677 39203519 PMC11356629

[40] Platts-Mills JA, Liu J, Rogawski ET, Kabir F, Lertsethtakarn P, Siguas M, et al. Use of quantitative molecular diagnostic methods to assess the aetiology, burden, and clinical characteristics of diarrhoea in children in low-resource settings: A reanalysis of the MAL-ED cohort study. Lancet Glob Health. 2018;6(12):e1309–18. doi: 10.1016/S2214-109X(18)30349-8 30287127 PMC6227251

[41] Das R, Haque MA, Chisti MJ, Faruque ASG, Ahmed T. Associated factors, post infection child growth, and household cost of invasive enteritis among under 5 children in Bangladesh. Sci Rep. 2021;11(1):12738. doi: 10.1038/s41598-021-92132-z 34140589 PMC8211821

[42] UNICEF, WHO, World Bank Group. Levels and trends in child malnutrition (2021 edition). 2021. [cited 2023 Feb 28]. Available from: https://data.unicef.org/resources/jme-report-2021/

[43] Schumacher D, Borghi E, Polonsky J, World Health Organization. Computation of the WHO Child Growth Standards: Package “anthro” in R. 2022. [cited 2023 Jan 15]. Available from: https://cran.r-project.org/web/packages/anthro/anthro.pdf

[44] World Health Organization. Childhood Stunting: Context, Causes and Consequences - Conceptual framework. 2016. [cited 2023 Mar 15]. Available from: https://www.who.int/publications/m/item/childhood-stunting-context-causes-and-consequences-framework

[45] Wirth JP, Rohner F, Petry N, Onyango AW, Matji J, Bailes A, et al. Assessment of the WHO Stunting Framework using Ethiopia as a case study. Matern Child Nutr. 2017;13(2):e12310. doi: 10.1111/mcn.12310 27126511 PMC6865882

[46] UNICEF. Conceptual Framework on Maternal and Child Nutrition, 2020. 2021. [cited 2023 Mar 15]. Available from: https://www.unicef.org/documents/conceptual-framework-nutrition

[47] World Health Organization. Improved sanitation facilities and drinking-water sources. [cited 2023 Mar 7]. Available from: https://www.who.int/data/nutrition/nlis/info/improved-sanitation-facilities-and-drinking-water-sources

[48] Ballard T, Coates J, Swindale A, Deitchler M. Household Hunger Scale: Indicator Definition and Measurement Guide. Washington, DC: Food and Nutrition Technical Assistance II Project, FHI 360.

[49] Checkley W, Buckley G, Gilman RH, Assis AM, Guerrant RL, Morris SS, et al. Multi-country analysis of the effects of diarrhoea on childhood stunting. Int J Epidemiol. 2008;37(4):816–30. doi: 10.1093/ije/dyn099 18567626 PMC2734063

[50] Ayana AB, Hailemariam TW, Melke AS. Determinants of acute malnutrition among children aged 6–59 months in Public Hospitals, Oromia region, West Ethiopia: A case–control study. BMC Nutr. 2015;1(1):1–11. doi: 10.1186/s40795-015-0031-9

[51] Rogawski McQuade ET, Shaheen N, Kabir F. Impact of Shigella infections and inflammation early in life on child growth and school-aged cognitive outcomes: Findings from three birth cohorts over 8 years. J Infect Dis. 2022;225(5):jiab434. doi: 10.1093/infdis/jiab434 PMC9534434 36149931

[52] Richard SA, Black RE, Gilman RH, Guerrant RL, Kang G, Lanata CF, et al. Catch-up growth occurs after diarrhea in early childhood. J Nutr. 2014;144(6):965–71. doi: 10.3945/jn.113.187161 24699805 PMC4018956

[53] Starkweather K, Keith M, Zohora FT, Alam N. Impacts of women’s work and childcare on child illness among Bangladeshi Shodagor communities. Soc Sci Med. 2024;359:117277. doi: 10.1016/j.socscimed.2024.117277 39217717

[54] Jakaria M, Bakshi RK, Hasan MM. Is maternal employment detrimental to children’s nutritional status? Evidence from Bangladesh. Rev Dev Econ. 2021;26(1):85–111. doi: 10.1111/rode.12819

[55] Rahman MdA, Rahman MdS, Shakur SM, Howlader MdH, Ashikuzzaman Md, Husna AU, et al. Risk factors of chronic childhood malnutrition: an analysis of the Bangladesh demographic and health survey 2014 data. J Public Health (Berl). 2020;30(2):309–21. doi: 10.1007/s10389-020-01281-4

[56] Islam MS, Biswas T. Prevalence and correlates of the composite index of anthropometric failure among children under 5 years old in Bangladesh. Matern Child Nutr. 2020;16(2):16. doi: 10.1111/MCN.12930 PMC7083426 31867876

[57] Victora CG, de Onis M, Hallal PC, Blössner M, Shrimpton R. Worldwide timing of growth faltering: Revisiting implications for interventions. Pediatrics. 2010;125(3):e473-80. doi: 10.1542/peds.2009-1519 20156903

[58] Dangour AD, Watson L, Cumming O, Boisson S, Che Y, Velleman Y, et al. Interventions to improve water quality and supply, sanitation and hygiene practices, and their effects on the nutritional status of children. Cochrane Database Syst Rev. 2013;2013(8):CD009382. doi: 10.1002/14651858.CD009382.pub2 23904195 PMC11608819

[59] Luby SP, Rahman M, Arnold BF, Unicomb L, Ashraf S, Winch PJ, et al. Effects of water quality, sanitation, handwashing, and nutritional interventions on diarrhoea and child growth in rural Bangladesh: A cluster randomised controlled trial. Lancet Glob Health. 2018;6(3):e302–15. doi: 10.1016/S2214-109X(17)30490-4 29396217 PMC5809718

[60] Budge S, Parker AH, Hutchings PT, Garbutt C. Environmental enteric dysfunction and child stunting. Nutr Rev. 2019;77(4):240–53. doi: 10.1093/nutrit/nuy068 30753710 PMC6394759

[61] Rah JH, Cronin AA, Badgaiyan B, Aguayo VM, Coates S, Ahmed S. Household sanitation and personal hygiene practices are associated with child stunting in rural India: A cross-sectional analysis of surveys. BMJ Open. 2015;5(2):e005180. doi: 10.1136/bmjopen-2014-005180 25678539 PMC4330332

[62] de Wit CC, Sas TCJ, Wit JM, Cutfield WS. Patterns of catch-up growth. J Pediatr. 2013;162(2):415–20. doi: 10.1016/j.jpeds.2012.10.014 23153864

[63] George CM, Perin J, Parvin T, Bhuyian MSI, Thomas ED, Monira S, et al. Diarrhea prevalence and child growth faltering are associated with subsequent adverse child developmental outcomes in Bangladesh (CHoBI7 Program). Am J Trop Med Hyg. 2021;106(1):233–8. doi: 10.4269/ajtmh.21-0767 34724631 PMC8733517

[64] Hati EP, Yani A, Paramashanti B, Rum FF. Maternal education and its impact on childhood stunting in low income settings. NutriSehat: Jurnal Ilmu Gizi. 2025;1(1):44–57.

[65] Lamberti LM, Fischer Walker CL, Black RE. Systematic review of diarrhea duration and severity in children and adults in low- and middle-income countries. BMC Public Health. 2012;12:276. doi: 10.1186/1471-2458-12-276 22480268 PMC3364857

[66] Richard SA, Black RE, Gilman RH, Guerrant RL, Kang G, Lanata CF, et al. Catch-up growth occurs after diarrhea in early childhood. J Nutr. 2014;144(6):965–71. doi: 10.3945/jn.113.187161 24699805 PMC4018956

[67] Richard SA, McCormick BJJ, Murray-Kolb LE, Bessong P, Shrestha SK, Mduma E, et al. Influences on catch-up growth using relative versus absolute metrics: Evidence from the MAL-ED cohort study. BMC Public Health. 2021;21(1):1246. doi: 10.1186/s12889-021-11120-0 34187407 PMC8240385

[68] Briend A, Hasan KZ, Aziz KM, Hoque BA. Are diarrhoea control programmes likely to reduce childhood malnutrition? Observations from rural Bangladesh. Lancet. 1989;2(8658):319–22. doi: 10.1016/s0140-6736(89)90498-4 2569114

[69] Schnee AE, Haque R, Taniuchi M, Uddin MJ, Alam MM, Liu J, et al. Identification of etiology-specific diarrhea associated with linear growth faltering in Bangladeshi infants. Am J Epidemiol. 2018;187(10):2210–8. doi: 10.1093/aje/kwy106 29767678 PMC6166216

[70] Black RE, Brown KH, Becker S. Effects of diarrhea associated with specific enteropathogens on the growth of children in rural Bangladesh. Pediatrics. 1984;73(6):799–805. doi: 10.1542/peds.73.6.799 6374599

[71] Bray AE, Ahmed S, Das SK, Khan SH, Chisti MJ, Ahmed T, et al. Viral pathogen-specific clinical and demographic characteristics of children with moderate-to-severe diarrhea in rural Bangladesh. Am J Trop Med Hyg. 2019;101(2):304–9. doi: 10.4269/ajtmh.19-0152 31264563 PMC6685579

[72] Sharif N, Nobel NU, Sakib N, Liza SM, Khan ST, Billah B, et al. Molecular and epidemiologic analysis of diarrheal pathogens in children with acute gastroenteritis in Bangladesh during 2014-2019. Pediatr Infect Dis J. 2020;39(7):580–5. doi: 10.1097/INF.0000000000002637 32187137

