# Supplementary material for: Factors influencing post-diarrhea growth patterns among children under two years old in rural Bangladesh
Source: PLoS Negl Trop Dis. 2026 Nov 5;20(11):e0014699. doi: 10.1371/journal.pntd.0014699 (PMC13754220; doi:10.1371/journal.pntd.0014699)
Supplement: Supplementary file 1 [file pntd.0014699.s001.docx]

S1 Table. Clinical risk factors associated with the crude change in LAZ, WAZ, WLZ, and MUAC during study follow-up among children under 24 months of age with watery diarrhea seeking care at Kumudini Hospital in Bangladesh

|  | **ΔLAZ** | | **ΔWAZ** | | **ΔWLZ** | | **ΔMUAC** | |
| --- | --- | --- | --- | --- | --- | --- | --- | --- |
|  | **Crude Difference (95% CI)^ad^** | **p-val^d^** | **Crude Difference (95% CI)^ad^** | **p-val^d^** | **Crude Difference (95% CI)^ad^** | **p-val^d^** | **Crude Difference (95% CI)^ad^** | **p-val^d^** |
| **Clinical Factors** | | | | | | | | |
| **Modified Vesikari Severity Score** | | | | | | | | |
| Mild (≤ 5) | ref |  | ref |  | ref |  | ref |  |
| Moderate/Severe (≥ 6) | -0.016  (-0.107, 0.068) | 0.665 | -0.013  (-0.145, 0.119) | 0.845 | -0.001  (-0.203, 0.200) | 0.989 | -0.005  (-0.089, 0.079) | 0.914 |
| **Number of loose stools within 24 hours before presentation** | | | | | | | | |
| ≤ 6 | ref |  | ref |  | ref |  | ref |  |
| 7-9 | 0.013  (-0.085, 0.110) | 0.799 | -0.006  (-0.165, 0.154) | 0.945 | -0.009  (-0.248, 0.230) | 0.939 | 0.010  (-0.076, 0.095) | 0.826 |
| ≥ 10 | -0.014  (-0.125, 0.097) | 0.806 | 0.060  (-0.109, 0.229) | 0.480 | 0.101  (-0.160, 0.362) | 0.444 | 0.022  (-0.092, 0.136) | 0.699 |
| Time* ≥ 10 | -0.039  (-0.096, 0.018) | 0.181 | 0.001  (-0.059, 0.062) | 0.967 | 0.020  (-0.067, 0.108) | 0.646 | 0.011  (-0.046, 0.069) | 0.697 |
| **Diarrhea duration of index case from onset (in days)** | | | | | | | | |
| 0-3 | ref |  | ref |  | ref |  | ref |  |
| 3-5 | -0.016  (-0.115, 0.084) | 0.758 | -0.082  (-0.249, 0.085) | 0.332 | -0.120  (-0.346, 0.106) | 0.293 | 0.034  (-0.080, 0.148) | 0.551 |
| 5-7 | -0.028  (-0.161, 0.106) | 0.680 | -0.195  (-0.245, 0.036) | 0.096 | -0.274  (-0.653, 0.105) | 0.153 | 0.023  (-0.118, 0.163) | 0.746 |
| ≥ 7 | **-0.163**  **(-0.284, -0.041)** | **0.010** | -0.060  (-0.246, 0.125) | 0.519 | 0.077  (-0.183, 0.337) | 0.557 | -0.058  (-0.165, 0.049) | 0.283 |
| Time*0-3 | 0.040  (-0.019, 0.098) | 0.181 | -0.022  (-0.082, 0.038) | 0.470 | -0.053  (-0.141, 0.036) | 0.240 | -0.059  (-0.126, 0.009) | 0.088 |
| Time*5-7 | 0.013  (-0.052, 0.079) | 0.687 | 0.023  (-0.051, 0.098) | 0.533 | 0.042  (-0.073, 0.157) | 0.468 | -0.052  (-0.126, 0.022) | 0.162 |
| Time* ≥ 7 | 0.055  (-0.004, 0.113) | 0.069 | -0.019  (-0.090, 0.053) | 0.604 | -0.075  (-0.178, 0.028) | 0.152 | -0.011  (-0.085, 0.063) | 0.765 |
| **Vomiting within 48 hours** | | | | | | | | |
| No | ref |  | ref |  | ref |  | ref |  |
| Yes | -0.045  (-0.145, 0.056) | 0.382 | 0.092  (-0.060, 0.244) | 0.233 | 0.167  (-0.082, 0.415) | 0.186 | 0.026  (-0.070, 0.122) | 0.594 |
| Time*Yes | - | - | **-0.055**  **(-0.108, -0.003)** | **0.039** | **-0.088**  **(-0.168, -0.008)** | **0.031** | **-** | **-** |
| **Fever** |  |  |  |  |  |  |  |  |
| No | ref |  | ref |  | ref |  | ref |  |
| Yes | 0.0009  (-0.084, 0.103) | 0.840 | -0.020  (-0.168, 0.128) | 0.793 | -0.045  (-0.270, 0.180) | 0.696 | -0.018  (-0.104, 0.069) | 0.690 |
| **Hospitalization at enrollment for diarrhea** | | | | | | | | |
| No | ref |  | ref |  | ref |  | ref |  |
| Yes | -0.038  (-0.118, 0.043) | 0.353 | -0.176  (-0.359, 0.007) | 0.059 | -0.201  (-0.464, 0.063) | 0.132 | **-0.137**  **(-0.234, -0.040)** | **0.007** |
| Time*Yes | -0.041  (-0.084, 0.002) | 0.059 | 0.055  (-0.003, 0.112) | 0.064 | **0.089**  **(0.003, 0.174)** | **0.044** | **-** | **-** |
| **Anorexia** |  |  |  |  |  |  |  | |
| No | ref |  | ref |  | ref |  | ref |  |
| Yes | 0.020  (-0.069, 0.110) | 0.654 | 0.047  (-0.086, 0.180) | 0.484 | 0.040  (-0.165, 0.245) | 0.699 | 0.050  (-0.037, 0.137) | 0.256 |
| Time*Yes | - | - | - | - | - | - | **-0.051**  **(-0.101, -0.002)** | **0.043** |

^a^Estimates of the best-fitting linear mixed-effects models, i.e., the random intercept and random slope models, based on AIC and BIC. Model comparison was based on Maximum Likelihood estimated models, while the reported coefficients and 95% confidence intervals (CIs) of the final model was obtained from Restricted Maximum Likelihood estimation.

^b^An interaction term of the risk factor with time was included only when a p-Value of <0.05 in the Likelihood Ratio Test comparing the full model with the interaction term and the nested model without the interaction term.

^c^Risk factors or their interaction terms with the time that had a p-Value of <0.20 in the bivariate analysis were included in the multivariate model.

^d^95% CIs of the risk differences and the p-Values were based on cluster-robust variance estimation with CR2 small sample correction.

Abbreviations: MUAC – mid-upper arm circumference; CI – confidence interval; p-val – p-Value; ref – reference

Fixed effects, statistically significant results (p<0.05) and borderline significant results (p < 0.10) are bold
