# Supplementary material for: Factors influencing post-diarrhea growth patterns among children under two years old in rural Bangladesh
Source: PLoS Negl Trop Dis. 2026 Nov 5;20(11):e0014699. doi: 10.1371/journal.pntd.0014699 (PMC13754220; doi:10.1371/journal.pntd.0014699)
Supplement: Supplementary file 2 [file pntd.0014699.s002.docx]

S2 Table. Sociodemographic factors associated with the crude change in LAZ, WAZ, WLZ, and MUAC during study follow-up among children under 24 months of age with watery diarrhea seeking care at Kumudini Hospital in Bangladesh

|  | ΔLAZ | | ΔWAZ | | ΔWLZ | | ΔMUAC | |
| --- | --- | --- | --- | --- | --- | --- | --- | --- |
|  | Crude Difference (95% CI)^ad^ | p-val^d^ | Crude Difference (95% CI)^ad^ | p-val^d^ | Crude Difference (95% CI)^ad^ | p-val^d^ | Crude Difference (95% CI)^ad^ | p-val^d^ |
| **Sociodemographic Factors** | | | | | | | | |
| **Baseline age (months)** | | | | | | | | |
| ≤ 6 | ref |  | ref |  | ref |  | ref |  |
| 7-12 | **0.161**  **(0.043, 0.278)** | **0.008** | 0.042  (-0.110, 0.194) | 0.587 | -0.096  (-0.334, 0.141) | 0.423 | **0.114**  **(0.005, 0.224)** | **0.042** |
| 13-24 | **0.113**  **(0.015, 0.211)** | **0.025** | 0.134  (-0.039, 0.307) | 0.128 | 0.045  (-0.227, 0.316) | 0.745 | **0.130**  **(0.036, 0.224)** | **0.007** |
| Time*7-12^b^ | **-** | **-** | **-0.068**  **(-0.125, -0.010)** | **0.022** | - | - | **-0.113**  **(-0.177, -0.049)** | **<0.001** |
| Time*13-24^b^ | **-** | **-** | **-0.096**  **(-0.151, -0.042)** | **<0.001** | -0.075  (-0.165, 0.015) | 0.101 | **-0.104**  **(-0.156, -0.053)** | **<0.001** |
| **Sex** |  |  |  |  |  |  |  |  |
| Male | ref |  | ref |  | ref |  | ref |  |
| Female | 0.067  (-0.022, 0.155) | 0.136 | -0.018  (-0.148, 0.112) | 0.786 | -0.09  (-0.293, 0.113) | 0.38 | 0.045  (-0.038, 0.128) | 0.288 |
| Time*Female | **-** | **-** | **-** | **-** | **-** | **-** | -0.034  (-0.081, 0.014) | 0.16 |
| **Head of household sex** | | | | | | | | |
| Male | ref |  | ref |  | ref |  | ref |  |
| Female | 0.025  (-0.083, 0.134) | 0.645 | 0.049  (-0.086, 0.182) | 0.476 | 0.04  (-0.146, 0.226) | 0.669 | 0.075  (-0.024, 0.174) | 0.133 |
| **Head of household education** | | | | | | | | |
| Illiterate, no education, incomplete primary | ref |  | ref |  | ref |  | ref |  |
| Completed primary, completed secondary | **0.097**  **(0.002, 0.192)** | **0.046** | -0.089  (-0.241, 0.063) | 0.248 | -0.225  (-0.463, 0.014) | 0.064 | **0.101**  **(0.001, 0.201)** | **0.048** |
| Completed higher education | **-0.153**  **(-0.278, -0.027)** | **0.019** | -0.059  (-0.260, 0.143) | 0.552 | 0.032  (-0.283, 0.347) | 0.834 | -0.088  (-0.185, 0.010) | 0.075 |
| Time*Completed primary, completed secondary | -0.041  (-0.091, 0.009) | 0.105 | 0.038  (-0.016, 0.093) | 0.168 | **0.093**  **(0.010, 0.176)** | **0.029** | -0.012  (-0.070, 0.045) | 0.674 |
| Time*Completed higher education | **0.079**  **(0.011, 0.147)** | **0.025** | -0.003  (-0.069, 0.064) | 0.930 | -0.060  (-0.154, 0.034) | 0.198 | 0.016  (-0.037, 0.069) | 0.544 |
| **Mother’s education** | | | | | | | | |
| Illiterate, no education, incomplete primary | ref |  | ref |  | ref |  | ref |  |
| Completed primary, completed secondary | -0.025  (-0.165, 0.114) | 0.716 | 0.014  (-0.189, 0.217) | 0.892 | 0.047  (-0.288, 0.381) | 0.779 | -0.097  (-0.239, 0.045) | 0.176 |
| Completed higher education | -0.066  (-0.213, 0.081) | 0.371 | 0.005  (-0.214, 0.224) | 0.963 | 0.063  (-0.304, 0.429) | 0.733 | -0.137  (-0.283, 0.010) | 0.067 |
| Time*Completed primary, completed secondary | 0.054  (-0.011, 0.118) | 0.100 | 0.010  (-0.056, 0.076) | 0.757 | -0.025  (-0.131, 0.082) | 0.640 | **0.064**  **(0.002, 0.126)** | **0.042** |
| Time*Completed higher education | 0.044  (-0.025, 0.114) | 0.205 | 0.009  (-0.063, 0.082) | 0.793 | -0.011  (-0.126, 0.105) | 0.856 | 0.060  (-0.003, 0.122) | 0.060 |
| **Monthly household income (Taka)** | | | | | | | | |
| ≤ 20,000 | ref |  | ref |  | ref |  | ref |  |
| > 20,000 | -0.021  (-0.111, 0.068) | 0.641 | 0.007  (-0.126, 0.140) | 0.913 | 0.018  (-0.184, 0.220) | 0.860 | -0.012  (-0.097, 0.074) | 0.789 |
| **Feeding Type** | | | | | | | | |
| Exclusive or partial breastfeeding | ref |  | ref |  | ref |  | ref |  |
| Supplemental feeding only | -0.038  (-0.192, 0.117) | 0.619 | -0.085  (-0.295, 0.125) | 0.409 | -0.054  (-0.362, 0.254) | 0.720 | -0.088  (-0.229, 0.054) | 0.211 |
| **Household Ratio (# of people living in household/# of bedrooms in household)** | | | | | | | | |
| 1-3 people per bedroom | ref |  | ref |  | ref |  |  |  |
| 3-4 people per bedroom | **-0.129**  **(-0.241, -0.017)** | **0.025** | -0.038  (-0.206, 0.130) | 0.651 | 0.073  (-0.169, 0.315) | 0.547 | -0.076  (-0.171, 0.019) | 0.112 |
| 4-5 people per bedroom | 0.019  (-0.109, 0.148) | 0.755 | -0.226  (-0.462, 0.009) | 0.059 | -0.299  (-0.609, 0.011) | 0.058 | -0.066  (-0.186, 0.054) | 0.264 |
| Time*3-4 people per bedroom | 0.048  (-0.014. 0.110) | 0.124 | - | - | - | - | - | - |
| Time*4-5 people per bedroom | - | - | 0.071  (-0.016, 0.158) | 0.104 | - | - | - | - |

^a^Estimates of the best-fitting linear mixed-effects models, i.e., the random intercept and random slope models, based on AIC and BIC. Model comparison was based on Maximum Likelihood estimated models, while the reported coefficients and 95% confidence intervals (CIs) of the final model was obtained from Restricted Maximum Likelihood estimation.

^b^An interaction term of the risk factor with time was included only when a p-Value of <0.05 in the Likelihood Ratio Test comparing the full model with the interaction term and the nested model without the interaction term.

^c^Risk factors or their interaction terms with the time that had a p-Value of <0.20 in the bivariate analysis were included in the multivariate model.

^d^95% CIs of the risk differences and the p-Values were based on cluster-robust variance estimation with CR2 small sample correction.

Abbreviations: MUAC – mid-upper arm circumference; CI – confidence interval; p-val – p-Value; ref – reference

Fixed effects, statistically significant results (p<0.05) and borderline significant results (p < 0.10) are bold
