# Supplementary material for: Factors influencing post-diarrhea growth patterns among children under two years old in rural Bangladesh
Source: PLoS Negl Trop Dis. 2026 Nov 5;20(11):e0014699. doi: 10.1371/journal.pntd.0014699 (PMC13754220; doi:10.1371/journal.pntd.0014699)
Supplement: Supplementary file 3 [file pntd.0014699.s003.docx]

S3 Table. Sanitation and Hygiene risk factors associated with the crude change in LAZ, WAZ, WLZ, and MUAC during study follow-up among children under 24 months of age with watery diarrhea seeking care at Kumudini Hospital in Bangladesh

|  | ΔLAZ | | ΔWAZ | | ΔWLZ | | ΔMUAC | |
| --- | --- | --- | --- | --- | --- | --- | --- | --- |
|  | Crude Difference (95% CI)^ad^ | p-val^d^ | Crude Difference (95% CI)^ad^ | p-val^d^ | Crude Difference (95% CI)^ad^ | p-val^d^ | Crude Difference (95% CI)^ad^ | p-val^d^ |
| **Sanitation and Hygiene Factors** | | | | | | | | |
| **Handwashing with soap before prepping food** | | | | | | | | |
| No | ref |  | ref |  | ref |  | ref |  |
| Sometimes or consistently | 0.046  (-0.050, 0.141) | 0.340 | -0.038  (-0.177, 0.101) | 0.583 | -0.095  (-0.287, 0.098) | 0.328 | 0.025  (-0.065, 0.116) | 0.574 |
| Time*Sometimes or consistently | -0.008  (-0.063, 0.046) | 0.756 | 0.008  (-0.050, 0.066) | 0.781 | 0.022  (-0.057, 0.100) | 0.580 | -0.034  (-0.100, 0.032) | 0.310 |
| **Handwashing with soap before feeding children** | | | | | | | | |
| No | ref |  | ref |  | ref |  | ref |  |
| Sometimes or consistently | **0.155**  **(0.045, 0.266)** | **0.009** | -0.031  (-0.235, 0.173) | 0.750 | -0.172  (-0.444, 0.101) | 0.201 | -0.013  (-0.128, 0.102) | 0.813 |
| Time*Sometimes or consistently | -0.057  (-0.148, 0.035) | 0.206 | 0.037  (-0.039, 0.113) | 0.318 | 0.101  (-0.004, 0.205) | 0.058 | 0.010  (-0.063, 0.083) | 0.774 |
| **Toilet facility** | | | | | | | | |
| Unimproved | ref |  | ref |  | ref |  | ref |  |
| Improved | 0.063  (-0.042, 0.168) | 0.236 | -0.094  (-0.244, 0.056) | 0.217 | -0.182  (-0.411, 0.047) | 0.117 | 0.011  (-0.073, 0.095) | 0.794 |
| Time*Improved^b^ | - | - | **0.063**  **(0.012, 0.115)** | **0.017** | **0.109**  **(0.030, 0.188)** | **0.007** | **-** | - |
| **Disposal of child’s stool** | | | | | | | | |
| Latrine/Toilet | **ref** |  | **ref** |  | **ref** |  | **ref** |  |
| Thrown in the garbage | 0.037  (-0.063, 0.138) | 0.461 | -0.041  (-0.210, 0.128) | 0.630 | -0.088  (-0.346, 0.169) | 0.497 | 0.002  (-0.092, 0.095) | 0.969 |
| Buried | 0.078  (-0.81, 0.237) | 0.235 | 0.022  (-0.0281, 0.325) | 0.845 | -0.032  (-0.425, 0.361) | 0.825 | -0.062  (-0.492, 0.368) | 0.697 |
| Thrown in ditch/drain/pond | -0.003  (-0.119, 0.114) | 0.960 | -0.042  (-0.223, 0.139) | 0.645 | -0.028  (0.302, 0.246) | 0.838 | **-0.104**  **(-0.207, -0.002)** | **0.046** |
| **Handwashing with soap after using toilet facility** | | | | | | | | |
| No | ref |  | ref |  | ref |  | ref |  |
| Yes | 0.148  (-0.121, 0.416) | 0.146 | 0.041  (-0.122, 0.203) | 0.408 | -0.076  (-0.436, 0.285) | 0.473 | -0.024  (-0.380, 0.332) | 0.804 |
| Time*Yes | -0.094  (-0.289, 0.101) | 0.180 | 0.048  (-0.279, 0.375) | 0.604 | 0.177  (-0.455, 0.810) | 0.362 | -0.045  (-0.388, 0.297) | 0.637 |
| **Handwashing with soap after cleaning children’s bottoms** | | | | | | | | |
| No | ref |  | ref |  | ref |  | ref |  |
| Sometimes or consistently | 0.045  (-0.137, 0.226) | 0.540 | 0.097  (-0.209, 0.402) | 0.438 | 0.071  (-0.368, 0.510) | 0.683 | -0.021  (-0.195, 0.153) | 0.759 |
| Time*Sometimes or consistently | 0.001  (-0.153, 0.156) | 0.983 | 0.049  (-0.227, 0.325) | 0.653 | 0.111  (-0.267, 0.490) | 0.469 | -0.030  (-0.240, 0.180) | 0.717 |
| **Type of handwashing facility in the household** | | | | | | | | |
| Tap or faucet | ref |  | ref |  | ref |  | ref |  |
| Basin or bucket | **-0.078**  **(-0.142, -0.014)** | **0.017** | **-0.618**  **(-0.713, -0.522)** | **<0.001** | **-0.74**  **(-0.889, -0.590)** | **<0.001** | **-0.300**  **(-0.359, -0.241)** | **<0.001** |
| Tube well | 0.029  (-0.060, 0.118) | 0.518 | 0.032  (-0.101, 0.165) | 0.637 | 0.028  (-0.174, 0.230) | 0.785 | 0.026  (-0.059, 0.110) | 0.55 |
| Time*Basin or bucket | **-0.05**  **(-0.084, -0.015)** | **0.006** | - | - | 0.048  (-0.007, 0.102) | 0.085 | - | - |
| Time*Tube well | -0.03  (-0.074, 0.015) | 0.19 | - | - | - | - | - | - |
| **Main source of drinking water** | | | | | | | | |
| Piped/tap water | ref |  | ref |  | ref |  | ref |  |
| Tube well | 0.021  (-0.069, 0.111) | 0.644 | 0.064  (-0.071, 0.199) | 0.351 | 0.081  (-0.126, 0.288) | 0.441 | 0.029  (-0.057, 0.115) | 0.501 |
| Time*Tube well | -0.04  (-0.086, 0.006) | 0.089 | - | - | - | - | - | - |
| **How drinking water is stored** | | | | | | | | |
| No storage of water | ref |  | ref |  | ref |  | ref |  |
| Closed Vessels | 0.008  (-0.101, 0.118) | 0.879 | -0.015  (-0.210, 0.180) | 0.879 | -0.055  (-0.342, 0.232) | 0.702 | 0.067  (-0.052, 0.186) | 0.264 |
| Open Vessels | 0.005  (-0.136, 0.147) | 0.939 | -0.095  (-0.363, 0.173) | 0.478 | -0.161  (-0.557, 0.235) | 0.417 | 0.025  (-0.105, 0.156) | 0.698 |
| **Treatment of drinking water** | | | | | | | | |
| Not treated | ref |  | ref |  | ref |  | ref |  |
| Treated (Boiled, Filtered) | -0.099  (-0.253, 0.054) | 0.174 | -0.050  (-0.241, 0.141) | 0.560 | -0.050  (-0.346, 0.338) | 0.560 | -0.017  (-0.107, 0.073) | 0.676 |
| Time*Treated (Boiled, Filtered) | 0.066  (-0.013, 0.144) | 0.089 | 0.076  (-0.23, 0.175) | 0.113 | 0.076  (-0.080, 0.188) | 0.113 | 0.04  (-0.059, 0.138) | 0.380 |
| **Source of bathing water** | | | | | | | | |
| Piped/tap water | ref |  | ref |  | ref |  | ref |  |
| Tube well | -0.003  (-0.091, 0.085) | 0.947 | 0.037  (-0.097, 0.172) | 0.584 | 0.064  (-0.139, 0.266) | 0.536 | -0.005  (-0.087, 0.078) | 0.910 |
| **River** | **0.310**  **(0.245, 0.374)** | **<0.001** | 0.013  (-0.079, 0.105) | 0.773 | **-0.252**  **(-0.397, -0.106)** | **<0.001** | **0.19**  **(0.125, 0.254)** | **<0.001** |
| **Time*River** | **-0.084**  **(-0.118, -0.050)** | **<0.001** | **-0.04**  **(-0.073, -0.008)** | **0.015** | 0.036  (-0.015, 0.088) | 0.163 | **-0.126**  **(-0.160, -0.092)** | **<0.001** |
| **Parental knowledge on the prevention of diarrhea** | | | | | | | | |
| ≤ 2 correct answers of 8 | ref |  | ref |  | ref |  | ref |  |
| ≥ 3 correct answers of 8 | 0.086  (-0.011, 0.184) | 0.082 | -0.009  (-0.147, 0.130) | 0.901 | -0.073  (-0.289, 0.144) | 0.506 | 0.053  (-0.037, 0.144) | 0.244 |
| **Own any animal** | | | | | | | | |
| No | ref |  | ref |  | ref |  | ref |  |
| Yes | -0.069  (-0.209, 0.071) | 0.326 | 0.019  (-0.167, 0.204) | 0.840 | 0.095  (-0.213, 0.404) | 0.536 | -0.096  (-0.208, 0.017) | 0.093 |
| Time*Yes | - | - | - | - | - | - | 0.043  (-0.016, 0.101) | 0.147 |

^a^Estimates of the best-fitting linear mixed-effects models, i.e., the random intercept and random slope models, based on AIC and BIC. Model comparison was based on Maximum Likelihood estimated models, while the reported coefficients and 95% confidence intervals (CIs) of the final model was obtained from Restricted Maximum Likelihood estimation.

^b^An interaction term of the risk factor with time was included only when a p-Value of <0.05 in the Likelihood Ratio Test comparing the full model with the interaction term and the nested model without the interaction term.

^c^Risk factors or their interaction terms with the time that had a p-Value of <0.20 in the bivariate analysis were included in the multivariate model.

^d^95% CIs of the risk differences and the p-Values were based on cluster-robust variance estimation with CR2 small sample correction.

Abbreviations: MUAC – mid-upper arm circumference; CI – confidence interval; p-val – p-Value; ref – reference

Fixed effects, statistically significant results (p<0.05) and borderline significant results (p < 0.10) are bold
