# Supplementary material for: Factors influencing post-diarrhea growth patterns among children under two years old in rural Bangladesh
Source: PLoS Negl Trop Dis. 2026 Nov 5;20(11):e0014699. doi: 10.1371/journal.pntd.0014699 (PMC13754220; doi:10.1371/journal.pntd.0014699)
Supplement: Supplementary file 4 [file pntd.0014699.s004.docx]

S4 Table. Food Security risk factors associated with the crude change in LAZ, WAZ, WLZ, and MUAC during study follow-up among children under 24 months of age with watery diarrhea seeking care at Kumudini Hospital in Bangladesh

|  | ΔLAZ | | ΔWAZ | | ΔWLZ | | ΔMUAC | |
| --- | --- | --- | --- | --- | --- | --- | --- | --- |
|  | Crude Difference (95% CI)^ad^ | p-val^d^ | Crude Difference (95% CI)^ad^ | p-val^d^ | Crude Difference (95% CI)^ad^ | p-val^d^ | Crude Difference (95% CI)^ad^ | p-val^d^ |
| **Food Security Factors based on the last 6 months** | | | | | | | | |
| **Couldn’t buy enough food and didn’t have money to get more** | | | | | | | | |
| Sometimes True | ref |  | ref |  | ref |  | ref |  |
| Never True | -0.09  (-0.285, 0.105) | 0.295 | 0.177  (-0.130, 0.483) | 0.203 | 0.35  (-0.083, 0.780) | 0.094 | -0.05  (-0.305, 0.206) | 0.644 |
| Don’t know/Refused to Answer | **-0.253**  **(-0.444, -0.063)** | **0.019** | **0.937**  **(0.635, 1.238)** | **<0.001** | **1.509**  **(1.088, 1.930)** | **<0.001** | 0.043  (-0.213, 0.300) | 0.682 |
| Time*Don’t know/Refused to answer | 0.111  (-0.018, 0.239) | 0.077 | **-0.076**  **(-0.151, -0.001)** | **0.048** | -0.125 | 0.07 | - | - |
| **Couldn’t afford to eat balanced meals (meal that includes fish or meat)** | | | | | | | | |
| Often True | ref |  | ref |  | ref |  | ref |  |
| Sometimes True | **-0.132**  **(-0.259, -0.005)** | **0.042** | **-0.128**  **(-0.381, -0.054)** | **0.011** | -0.168  (-0.396, 0.059) | 0.139 | **-0.329**  **(-0.445, -0.213)** | **<0.001** |
| Never True | **-0.172**  **(-0.220, -0.124)** | **<0.001** | **-0.128**  **(-0.202, -0.054)** | **<0.001** | -0.018  (-0.132, 0.097) | 0.762 | **-0.346**  **(-0.391, -0.300)** | **<0.001** |
| Time*Sometimes true | **0.201**  **(0.134, 0.269)** | **<0.001** | **0.125**  **(0.047, 0.203)** | **0.003** | - | - | **0.184**  **(0.101, 0.268)** | **<0.001** |
| Time*Never true | **0.159**  **(0.135, 0.182)** | **<0.001** | **0.177**  **(0.152, 0.202)** | **<0.001** | **0.102**  **(0.064, 0.140)** | **<0.001** | **0.216**  **(0.191, 0.241)** | **<0.001** |
| **Cut the size of meals or skipped meals because there wasn’t enough money for food** | | | | | | | | |
| No | ref |  | ref |  | ref |  | ref |  |
| Some months but not every month | 0.135  (-0.075, 0.345) | 0.153 | -0.158  (-0.550, 0.234) | 0.334 | -0.365  (-0.924, 0.195) | 0.148 | 0.102  (-0.186, 0.390) | 0.39 |
| **Eaten less than what was needed because there wasn’t enough money for food** | | | | | | | | |
| No | ref |  | ref |  | ref |  | ref |  |
| Yes | 0.029  (-0.211, 0.269) | 0.662 | -0.087  (-1.092, 0.917) | 0.753 | -0.161  (-1.368, 1.046) | 0.635 | 0.006  (-0.645, 0.656) | 0.974 |
| Don’t Know | **0.201**  **(0.156, 0.246)** | **<0.001** | **-0.342**  **(-0.410, -0.275)** | **<0.001** | **-0.726**  **(-0.830, -0.623)** | **<0.001** | **0.293**  **(0.250, 0.335)** | **<0.001** |
| Time*Don’t know | **0.149**  **(0.126, 0.172)** | **<0.001** | **0.028**  **(0.003, 0.053)** | **0.027** | **-0.222**  **(-0.259, -0.185)** | **<0.001** | **0.101**  **(0.076, 0.126)** | **<0.001** |
| **Were hungry but didn’t eat because there wasn’t enough money for food** | | | | | | | | |
| No | ref |  | ref |  | ref |  | ref |  |
| Yes | 0.052  (-0.964, 1.068) | 0.649 | 0.055  (-4.032, 4.142) | 0.898 | 0  (-5.05, 5.05) | 1.00 | 0.137  (-1.603, 1.878) | 0.517 |
| **Household Hunger Scale** |  |  |  |  |  |  |  |  |
| Little to no hunger in household | ref |  | ref |  | ref |  | ref |  |
| Moderate hunger in household | 0.103  (-0.059, 0.265) | 0.173 | -0.136  (-0.410, 0.137) | 0.274 | -0.302  (-0.680, 0.076) | 0.100 | 0.094  (-0.137, 0.324) | 0.366 |
| Time*Moderate hunger in household | - | - | -0.054  (-0.135, 0.026) | 0.153 | - | - |  |  |

^a^Estimates of the best-fitting linear mixed-effects models, i.e., the random intercept and random slope models, based on AIC and BIC. Model comparison was based on Maximum Likelihood estimated models, while the reported coefficients and 95% confidence intervals (CIs) of the final model was obtained from Restricted Maximum Likelihood estimation.

^b^An interaction term of the risk factor with time was included only when a p-Value of <0.05 in the Likelihood Ratio Test comparing the full model with the interaction term and the nested model without the interaction term.

^c^Risk factors or their interaction terms with the time that had a p-Value of <0.20 in the bivariate analysis were included in the multivariate model.

^d^95% CIs of the risk differences and the p-Values were based on cluster-robust variance estimation with CR2 small sample correction.

Abbreviations: MUAC – mid-upper arm circumference; CI – confidence interval; p-val – p-Value; ref – reference

Fixed effects, statistically significant results (p<0.05) and borderline significant results (p < 0.10) are bold
